# LIN28 Family in Testis: Control of Cell Renewal, Maturation, Fertility and Aging

**DOI:** 10.3390/ijms23137245

**Published:** 2022-06-29

**Authors:** Dajana Krsnik, Tihana Marić, Floriana Bulić-Jakuš, Nino Sinčić, Ana Katušić Bojanac

**Affiliations:** 1 Scientific Centre of Excellence for Reproductive and Regenerative Medicine, School of Medicine, University of Zagreb, 10000 Zagreb, Croatia; dajana.krsnik@mef.hr (D.K.); tihana.maric@mef.hr (T.M.); floriana.bulic@mef.hr (F.B.-J.); nino.sincic@mef.hr (N.S.); 2 Department of Medical Biology, School of Medicine, University of Zagreb, 10000 Zagreb, Croatia

**Keywords:** LIN28A, LIN28B, testis, cancer, infertility

## Abstract

Male reproductive development starts early in the embryogenesis with somatic and germ cell differentiation in the testis. The LIN28 family of RNA-binding proteins promoting pluripotency has two members—LIN28A and LIN28B. Their function in the testis has been investigated but many questions about their exact role based on the expression patterns remain unclear. LIN28 expression is detected in the gonocytes and the migrating, mitotically active germ cells of the fetal testis. Postnatal expression of LIN28 A and B showed differential expression, with LIN28A expressed in the undifferentiated spermatogonia and LIN28B in the elongating spermatids and Leydig cells. LIN28 interferes with many signaling pathways, leading to cell proliferation, and it is involved in important testicular physiological processes, such as cell renewal, maturation, fertility, and aging. In addition, aberrant LIN28 expression is associated with testicular cancer and testicular disorders, such as hypogonadotropic hypogonadism and Klinefelter’s syndrome. This comprehensive review encompasses current knowledge of the function of LIN28 paralogs in testis and other tissues and cells because many studies suggest LIN28AB as a promising target for developing novel therapeutic agents.

## 1. Introduction

An unusually high incidence of male reproductive impairment and genital malformations has been reported in baby boys and young men in the last few decades. The reported male fertility rates are declining in the Western world, which further brings focus to male reproduction [1]. The basic molecular processes that promote sexual dimorphism and testis differentiation begin early in fetal development, with the somatic testicular cells (Sertoli and Leydig cells) as the leading sex determination drivers [2]. Sertoli cells then direct the development of early germline precursors into spermatogenic cell lines. These events have also been supported by the testosterone surge from interstitial Leydig cells. Hence, male reproductive health is highly dependent on the proper functioning of the molecular interplays in the early development of the testis, which can lead to early consequences already during the fetal period [3]. Recent research has suggested the vital role of the LIN28 protein family in testis function and spermatogenesis. LIN28 are RNA-binding proteins that support pluripotency by regulating the biogenesis of the microRNA let-7 through direct binding [4]. The heterochronic gene LIN28 was first discovered and studied in the nematode Caenorhabditis elegans, where it was required to control developmental timing [5]. In mammals, two members of the LIN28 family exist that produce LIN28A and LIN28B proteins. LIN28A and LIN28B paralogs show a high degree of homology in their structural domains with similar but still partly different functions [6]. The LIN28AB proteins exist and define “stemness” in pluripotent embryonic stem cells (ESCs) and germ cells through let-7 signaling [7]. In this review, we aimed to widely encompass current knowledge on the LIN28 role in testis development and health issues and the LIN28 signaling pathways in other tissues. Moreover, we discuss LIN28 as a possible molecular target for treating disorders with new strategies. 

## 2. Expression and Function of LIN28AB in Tissues

To understand the function of the LIN28 family in the testis, it is essential to form a broader picture of its presence in other tissues. Moreover, it is crucial to understand the dynamics of its temporal expression. Both LIN28A and LIN28B are highly expressed in human undifferentiated embryonic stem cells (hESCs), but a decrease in LIN28AB expression has been reported in human ESCs during differentiation [8,9]. It was shown that, in spontaneously differentiated hESCs, the mRNA levels of LIN28A and LIN28B were significantly lower but with slower kinetics of LIN28B downregulation than the reduction in LIN28A levels [9]. LIN28 is highly expressed in various fetal tissues and progenitor stem-like cells. However, its expression declines and is significantly downregulated with the developmental progression in most differentiated adult tissues [10], with some exceptions. LIN28 was found in neural progenitor cells of the mouse brain and was overexpressed in the neurons of the adult hippocampus, supporting neurogenesis, while it decreased during aging [11]. Such features seem to be tissue-dependent, as high LIN28A expression has recently been found in neonatal cardiac-tissue-derived stem-like cells (CTSCs) during heart development, but, in adult CTSCs, expression was low or absent [12]. High expression of LIN28B was detected in fetal hematopoietic stem and progenitor cells (HSPCs), but it was not confirmed in adult HSPCs [13].

Specific tissues persistently express LIN28, with the preference for A or B paralog. For example, high levels of LIN28AB are present in the trophoblast, but LIN28B is expressed almost 1300-fold higher than LIN28A [14,15,16,17]. High LIN28B expression was also detected in the placenta [18], where the LIN28B locus is imprinted, and paternal monoallelic expression is present in the human placental tissue [19]. Since LIN28 proteins inhibit cell differentiation and promote proliferation, their increased expression was detected in the placentas in the first trimester of pregnancy, compared to the term human placenta [20,21]. Accordingly, the induction of differentiation of mouse and human stem trophoblast cells was followed by a decrease in LIN28A expression [15]. Germline stem cells also retain high levels of LIN28. LIN28B expression remained high across gestation in the human fetal ovary, while LIN28A expression was highest in the early gonad containing only primordial germ cell (PGC) and decreased at later gestations, coincident with the onset of germ cell differentiation. Similarly, in the human male embryo at eight weeks of gestation (WGA), LIN28 was present in migrating primordial germ cells (PGCs) and early gonocytes [22]. However, its expression decreased later during further differentiation. Still, the LIN28AB expression remained specific for prespermatogonia (preSPG) of the early postnatal and spermatogonial stem cells (SSCs) of adult testis [22,23]. Lin28 mRNA is prominently expressed in the placenta, testis, ovary, and pituitary gland in rodents. Similarly, Lin28b mRNA is highly expressed in the adult rat testis and placenta. Transcripts of both Lin28a and Lin28b were also observed in the hypothalamus [18].

Both LIN28A and LIN28B were implicated as key factors in self-renewal and cell fate decisions, where they control the balance between the pluripotency and differentiation state. LIN28A, together with core pluripotency transcription factors OCT4, SOX2, and NANOG, is an important player in cellular reprograming, e.g., conversion of human somatic fibroblasts into self-renewing pluripotent stem cells (iPSCs) [24]. Similar to LIN28A, LIN28B can also regulate reprograming to pluripotency together with NANOG, OC4, and SOX2, while both LIN28A and LIN28B isoforms are required for maximum efficiency of reprograming in mouse and human PSCs. On the other hand, LIN28A/LIN28B deficiency reduces reprograming efficiency and arrests the derived mouse iPSCs in the naïve state. Furthermore, both LIN28A and LIN28B repress mitochondrial oxidative metabolism in mouse PSCs [25], which is characteristic of primed state pluripotency and higher LIN28B expression [26,27]. However, Zhang et al. showed differential regulation of these closely related paralogs in reprograming human fibroblasts, considering their kinetics and histone modifications of their promoters. LIN28B assumes an active chromatin structure and reactivates gene expression earlier in reprograming, while LIN28A is only upregulated upon acquisition of pluripotency [25]. *LIN28AB* genes are well-established regulators of developmental timing and vertebrate growth. Human genome-wide association studies (GWAS) have reported variants in the LIN28B gene implicated in features such as height [28,29,30], finger length ratio [31], adiposity [32], and also in the timing of puberty onset [33,34,35]. As all mentioned are traits associated with hormonal regulation, further studies on the zebrafish and human data showed that pubertal-timing-associated genetic variation correlates with the expression of LIN28B in the hypothalamus and pituitary gland [36]. However, the precise mechanisms of LIN28 action are not known, especially in humans. Some gene manipulation studies of LIN28A in transgenic mice showed that constitutive overexpression of LIN28A manifests in increased body size and delayed onset of puberty [37]. Fetal but not adult LIN28AB deficiency in knockout (KO) mice led to dwarfism and aberrations in glucose metabolism. LIN28A and LIN28B manifest important differences in the phenotypes mediated by their tissue and temporal-specific deficiency, as constitutive loss of LIN28A caused perinatal dwarfism and metabolic dysfunction. In contrast, constitutive loss of LIN28B did not influence metabolism until later in adults [38]. A recent study also supports distinct functions of LIN28A and LIN28B in cell fate decisions. It was shown that LIN28A or LIN28B overexpression induced nascent pro-mesodermal proliferative phenotype and increased caudal vertebrate number during tail development, while only LIN28A knockout caused the opposite effect [39]. Furthermore, LIN28A and LIN28B may have a distinct function in mammalian tooth development [40] or retinal progenitor cells (RPCs). In RPCs, only LIN28A regulates neurogliogenesis, in contrast to LIN28B, which did not influence the differentiation of RPCs [41]. 

The role of LIN28AB in female cells and tissues has also been explored. The study on C. elegans hermaphrodites showed that loss-of-function LIN28 mutants have oocytes that undergo DNA replication but neither ovulate nor fertilize [42]. The loss of LIN28A expression in the human trophoblast promoted the differentiation of syncytial cells. The LIN28 role appears to be inducing rather than actively initiating the differentiation of human trophoblast cells [15]. LIN28B knockdown showed that this protein is obligatory for maintaining cells in a progenitor-like state in the placenta. Moreover, when LIN28B was knocked out, the levels of chromatin-remodeling protein HMGA2 that maintains cell proliferation and growth during fetal and embryonal development were increased, probably due to increased LIN28A expression as a compensatory mechanism [43]. A recent study by Santoro et al. showed that circulating plasma miRNAs, identified as aberrantly expressed in pregnancy, target transcripts of proteins localized in the placenta, including LIN28B. This study indicates the importance of the proper miRNA expression and LIN28B regulation during pregnancy and its potential to disrupt trophoblast development genes essential for normal placenta development [44]. Double LIN28AB knockout in trophoblast cell lines leads to a decrease in the expression of proliferative markers and induction of differentiation [16,43,45]. The variety of LIN28AB roles is summarized in Table 1.

## 3. Structure and Subcellular Distribution of LIN28AB

Human LIN28AB genes are located at chromosomes 1p36.11 and 6q21. LIN28A gene encodes a 209-amino-acid protein, whereas LIN28B encodes a 250-amino-acid protein, but they show a high degree of homology in their structural domains. Both LIN28A and LIN28B exhibit highly conserved regions: the N-terminal cold-shock domain (CSD) and two C-terminal CysCysHisCys (CCHC) zinc finger or zinc knuckles domains (ZKD), which allow their RNA-binding function [6,7]. These RNA-binding domains are connected with a flexible linker, thereby accommodating LIN28 to bind different targets, mainly the members of let-7 miRNA [7]. The combination of one CSD and two CCHC zinc finger domains observed in LIN28AB protein was first found in plant RNA-binding protein GRP2, which is involved in the development, stress response, and genome organization [46]. Cold-shock domains are named after bacterial cold-shock proteins (CSPs) because they share the sequence motif characteristics. In bacteria, the CSD domain binds single-stranded nucleic acids. It enables CSPs to participate in regulating almost all steps of gene expression involving RNA, including transcription, translation, and RNA turnover. Retroviral-type CCHC zinc finger domain was first identified in the nucleocapsid protein (NCP) of the HIV-1 virus [47]. Unlike LIN28A, LIN28B has a nuclear localization signal (NLS) at the C-terminal end and a nucleolar localization signal (NoLS) between the CSD and first zinc finger domain [48]. These studies suggest LIN28B is a nuclear protein, but there are divergent opinions about LIN28B subcellular localization. One report showed that LIN28B is predominantly localized to the nucleus because of its NoLS and NLS fragments [48]. At the same time, others suggest that LIN28B is a cytoplasmic protein that may shuttle into the nucleus in a cell-cycle-dependent manner [6,49,50]. It seems that the subcellular location of LIN28B may vary depending on the cell type, but further studies are required to elucidate and validate these observations. In contrast, the subcellular localization of LIN28A is predominantly cytoplasmic, where it is involved in mRNA degradation, mRNA surveillance, translational repression, and gene silencing. Interestingly, LIN28A was shown to shuttle between the nucleus and cytoplasm under cellular stress, with localization in stress granules (SGs) and/or processing bodies (PBs) [51]. A study on the human fetal ovary demonstrated cell-differentiation-related change in the subcellular localization of the LIN28. In the early gonad, at nine weeks of gestational age (WGA), all germ cells showed both cytoplasmic and nuclear staining, but, at later gestation (18 WGA), most of the germ cells showed only cytoplasmic LIN28 [52]. Similarly, a study on human fetal and neonatal testis found both nuclear and cytoplasmic signals of LIN28 in gonocytes, while they were predominantly cytoplasmic in pre-spermatogonia [22].

The exact mechanism of nucleocytoplasmic shuttling of LIN28 is unknown. One study provides a possible model of the LIN28 nucleocytoplasmic shuttling, which includes RNA-binding protein Musashi1 (Msi1) and importin-α, a nuclear transport factor linking cNLS-containing proteins with importin-β. Accordingly, Msi1 can promote LIN28 nuclear import via importin-α subtype switching and its retention in the nucleus by enhancing the formation of LIN28-containing complexes [53]. Since importins (IMPs) play a pivotal role in the nuclear import [54,55], and because of their recently reviewed important role in spermatogenesis and male fertility [56], it would be useful to investigate the association between IMPs and nucleocytoplasmic shuttling of LIN28AB during male germ cells development. Additionally, epigenetic studies revealed a specific histone modification that contributes to the subcellular localization of LIN28A in the nucleus. Kim et al. found that the SET7/9, an exclusive H3K4 mono-methyltransferase, stabilizes LIN28 via lysine 135 (K135) methylation and contributes to nuclear retention of LIN28A. Furthermore, the K135 and its surrounding residues within LIN28A have sequential homology to the NoLS of LIN28B [57]. Like the NoLS of LIN28B, the SET7/9-mediated methylation of LIN28A may act as a switch that causes nuclear retention of LIN28A, which suggests that LIN28AB may share a specific role in the nucleoli. However, it remains unclear what induces LIN28 to shuttle into the nucleus and what its role is in that cellular compartment. Further studies are necessary to elucidate the mechanism of nucleocytoplasmic shuttling of LIN28A and LIN28B paralogs and their function inside the nucleus.

## 4. Expression Dynamics of LIN28 Family in the Testis

To understand the biological function and differential activities of LIN28 proteins in the testis, it is necessary to explore their expression dynamics through testicular development.

### 4.1. LIN28AB Expression Dynamics across Fetal Testis Development

**Figure 1:**
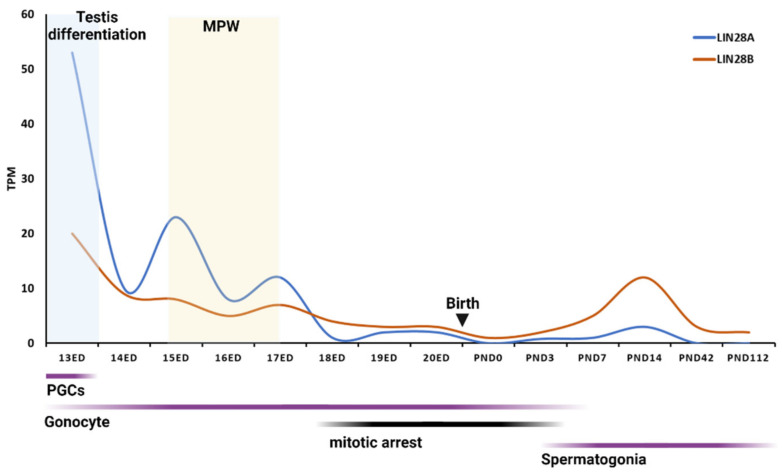
mRNA expression of Lin28ab across rat testis development. Expression levels of Lin28ab mRNAs are shown in transcripts per million (TPM). MPW—masculinization programing window. Data available free online [58,59]. Graph created with BioRender.com (accessed on 4 May 2022).

Similar LIN28AB expression patterns were observed during rodent and human prenatal testis development. In the mouse fetal testis, the highest expression of Lin28b was detected at 10.5 days post coitum (dpc), with a second peak (same as Lin28a) at 12.5 dpc [58,59]. At that time, mouse PGCs colonize the gonadal ridge (GR), and, around 12.5 dpc, sexual differentiation of the GR becomes apparent. At 13.5 dpc, mouse PGCs enter meiosis and begin to arrest [60]. Similarly, in rat fetal testis, Lin28a and Lin8b peak at 12 dpc and decline later in development [58,59]. PGCs give rise to mitotically active gonocytes but, during the mid-late fetal development, they enter a mitotic arrest that persists until a few days after birth, when mitotic activity resumes and gonocytes develop to spermatogonia, premeiotic spermatogenic cells [61]. Interestingly, gonocyte mitotic hiatus coincides with the lowest Lin28ab expression from GD 18 to PND 3 (Figure 1). 

**Figure 2:**
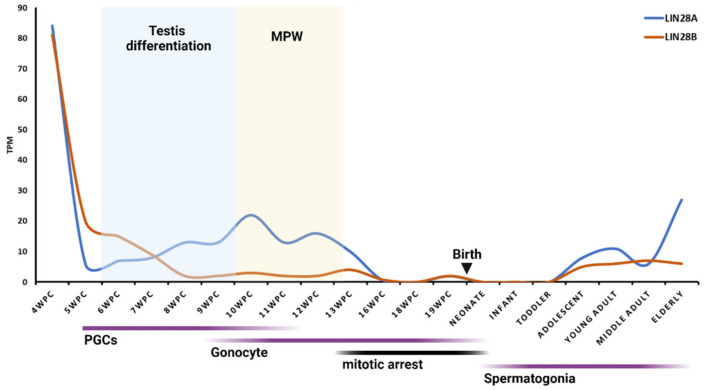
mRNA expression of *LIN28AB* across human testis development. Expression levels of *LIN28AB* mRNAs are shown in transcripts per million (TPM). MPW—masculinization programing window. Data available free online [58,59]. Graph created with BioRender.com (accessed on 4 May 2022).

Regarding humans, in extensive research of the transcriptome landscape of human primordial germ cells, the highest expression of LIN28A was detected at 4 WGA and low to medium expression from 7 to 19 WGA [62]. These results are in line with a recent study from Cardoso-Moreira et al. that found the highest expression of transcription factors, including both LIN28A and LIN28B, earlier in human testis development (4 WGA) [58,59]. That is the time of human primordial germ cell (PGCs) formation and migration when PGCs have been identified in the wall of the yolk sac and hindgut (for a review, see: [63]). LIN28AB expression decreases later in development [58,59] (Figure 2). The localization of LIN28B in the fetal testis is still unclear, while expression of LIN28A during human fetal testis development is restricted to gonocytes, germ cell precursors in fetal testis. Disorders in gonocytes’ function and differentiation during embryonal development can lead to germ cell neoplasia in situ (GCNIS), formerly called “Carcinoma in Situ (CIS)”. GCNIS is a precursor of most testicular germ cell tumors (TGCTs) [64,65], emphasizing the importance of researching the molecular factors involved in their development. During the first trimester (up to 12 WGA), gonocytes are mitotically active and form a quite homogenous population, both morphologically and histochemically. During the second trimester (from 12 WGA onwards), gonocytes progressively lose mitotic activity together with their pluripotency markers OCT4, VAS, NANOG, and c-KIT. Then, two new types of germ cells, besides gonocytes, are present, intermediate germ cells with proliferative capacity and mitotically quiescent prespermatogonia [66]. Recently, those types of cells have been confirmed by single-cell RNA sequencing (scRNA-seq). Around 15–16 WGA, primordial germ cells downregulate pluripotency-related genes, exit from the pluripotent-like state, and enter the G0 phase, followed by a transition into quiescent spermatogonia called “state f0” [67]. Single-cell RNA-seq analysis of human germline cells revealed high LIN28A expression in migrating and mitotic fetal germ cells and low LIN28A in arrested/mitotically quiescent germ cells [68]. These results may explain the declining expression of LIN28A from 13 to 19 WGA, although a pool of LIN28A-positive germ cells was still present in the testis [58,59] (Figure 2). Data about the expression dynamics of LIN28AB in human fetal testis from 19 WGA until birth are missing from the literature, probably due to hard-to-reach samples in such an advanced period of pregnancy. Nonetheless, regarding the germ cells’ mitotic arrest, we can assume that LIN28 expression is low in this period.

Interestingly, the LIN28 expression dynamics coincide with the masculinization programing window (MPW) and production of testosterone (T), both in rats and in humans (Figure 1 and Figure 2). In the rat fetal testis, MPW is reported to occur at gestational day 15.5 (GD 15.5) to GD 17.5 and it is directed by testosterone produced by fetal Leydig cells. The onset of T production is around 14.5 GD, peaks at 18 to 19 GD, and then declines immediately before birth. Human fetal testis becomes an endocrinologically active organ at 8 WG with the onset of T production. T peaks at WG 11 to 14 and declines at around WG 17 (for a review, see: [69]). Furthermore, a study found that LIN28KO male mice had lower plasma T levels and less frequent mating behavior than controls [70]. It would be interesting to further explore the potential link between LIN28AB and hormone (e.g., testosterone) signaling, as well as the effect of changing their expression during fetal period on possible changes in phenotype in postnatal life.

### 4.2. LIN28AB Expression Dynamics across Postnatal Testis Development

LIN28A and LIN28B mRNAs are present in the rodent and human testis across postnatal maturation, but their expression varies between neonatal and pubertal periods. In the postnatal testicular development of rodents, Lin28a peaks in the middle of the infantile period in both mice [58,59,71] and rats [4] (Figure 1). LIN28A expression declines during the puberty onset period in humans [23], rats [4], and mice [72]. A study from Cardoso-Moreira et al. detected a similar expression pattern for Lin28b [58] (Figure 1). However, other studies found the highest Lin28b expression around puberty [4,72]. 

Considering the cellular distribution, there are different expression patterns of LIN28A and LIN28B in the rodent postnatal testis. In human infancy, LIN28A expression was detectable in gonocytes and in undifferentiated and type A spermatogonia (SPG) and was absent from the testis interstitium [23]. The most recent scRNA-seq analysis of the neonatal human testis revealed the existence of three distinct germ cell states, named PGC-Like (PGCL), PreSPG-1, and PreSPG-2. Human fetal PGCs differentiate into PGCLs, which express the different gene and protein markers. PGCLs subsequently differentiate into PreSPGs [73], but whether the cellular distribution of LIN28 remains similar in these germ cell states remains unknown. On the other hand, LIN28B expression is restricted to the interstitium in both fetal and immature adult-type Leydig cell precursors (IALC) of neonatal/infantile mice. In contrast, no cells with LIN28B were found in the seminiferous tubules [4,23,72]. These results are in line with the study on marmoset monkey testes, where LIN28 was also detected within the interstitial compartment, most likely in Leydig cells (LC) [23]. The role of LIN28B in steroidogenic Leydig cells has not yet been described. Although different functional roles of LIN28A and LIN28B in the regulation of postnatal, prepubertal testis maturation in rodents are possible, additional studies are needed to elucidate LIN28B expression and function within the interstitial compartment of the human postnatal testis. 

RT-PCR analysis revealed that Lin28a and Lin28b are abundantly expressed in adult mouse testis. Immunohistochemical (IHC) analyses on the adult mouse testis showed that cells expressing LIN28A correspond to undifferentiated or A1 spermatogonia, as was the case in early postnatal and pubertal testis. LIN28B was also observed in the seminiferous tubules of the adult mouse testis, but, unlike LIN28A, it was expressed in the round and postmeiotic elongating spermatids. In addition, LIN28B was also detected in mature adult-type Leydig cells in the interstitial compartment of the adult mouse testis [72]. A similar expression profile of LIN28AB has been confirmed in the rat testis. It seems that LIN28AB and let-7 members of microRNA are mutually exclusive, as let-7b was expressed in pachytene spermatocytes that are negative for LIN28AB and absent from elongating spermatids which show high LIN28B expression in the mouse [4]. IHC analyses showed strong cytoplasmic staining of LIN28B in interstitial Leydig cells and nuclear staining within the seminiferous tubules, while LIN28A immunostaining was always cytoplasmic [72]. Interestingly, the expression of LIN28B in elongating spermatids coincides with expressional changes in key proteins involved in spermatid differentiation and maturational events in the male gonad. Such expressional changes are loss of histones and the appearance of a testis-specific HMG (tsHMG), histone H1-like protein in spermatids 1 (Hils1), transition proteins 1 and 2 (TP1 and TP2), and protamine 1, which all are the basic nuclear proteins [74,75]. These findings point to a possible role of LIN28B in regulating spermatid elongation events, likely via the let-7 regulatory mechanism.

Different data exist regarding the LIN28A expression in adult human testis. Gillis et al. detected neither LIN28A-positive germ cells after the first postnatal year nor throughout adulthood [22]. Aeckerle et al. revealed the presence of few LIN28A-positive spermatogonia in adult testis. Still, most tubules showed no LIN28A-positive spermatogonia [23]. As has been already mentioned, a novel scRNA-seq analysis of the adult human testis revealed distinct human SPG subsets, named spermatogonial stem cells 1 (SSC-1), spermatogonial stem cells 2 (SSC-2), early differentiating SPG (diff-SPG), and differentiating SPG (Diff-SPG). Based on the expression of specific marker genes, the SSC-1 subset is the most primitive and Diff-SPG is the most developmentally advanced [73]. These new findings enrich our view of male germ cell development. Like for the neonatal period, studies on the adult human testis are needed to investigate LIN28A localization in these different clusters of germ cells. Like the fetal and postnatal human testis, LIN28B expression has also been insufficiently investigated in adult human testis. Moreover, further studies are needed to confirm its presence and exact function in spermatids and Leydig cells of the human testis. Moreover, our knowledge about expression dynamics and hormonal regulation of this system in human testis is incomplete.

### 4.3. LIN28 in the Testicular Aging

One of the roles of the LIN28 family is to co-ordinate cellular growth and cellular metabolism to influence the metabolic physiology of aging, as reviewed by Jun-Hao et al. [76]. A study on mice showed that Lin28a/Lin28b/let-7 pathway is a key regulator of weight and previously mentioned pubertal timing in a sex-specific manner. Lin28a gain-of-function (GOF) caused heavier mice of both sexes, while Lin28b loss-of-function (LOF) led to lighter body weights only in male mice. Furthermore, Lin28a GOF mice resulted in the late onset of puberty in both sexes considering the beginning of puberty. At the same time, Lin28b LOF and let-7 GOF had a similar alteration in pubertal timing but only in male mice [77].

The role of LIN28 in the cellular aging process was shown in several studies on animal models. A recent study on Drosophila establishes that Lin28 has a pivotal role in the function and aging of the male germline stem cell niche. Lin28 is expressed in hub cells in early testis development, with the highest level in young adults, and then declines with age. Similarly, Lin28 mutations resulted in a loss of hub cell number, aberrant cell size, and impaired morphology. Importantly, it was found that re-expression of Lin28 in Lin28 mutant and maintaining the expression of Lin28 specifically in hub cells of the adult testis can preserve the number of hub cells and rescue the mutant phenotype. Furthermore, data demonstrated that LIN28 is capable of binding to and controlling the stability of the self-renewal factor unpaired (Upd) in hub cells and that the loss of LIN28 in the hub niche is associated with decreased Upd levels, hub cell aging, and loss of the stem cell niche. LIN28 directly binds to the sequence-specific motif (GGAGA) at the Upd mRNA 3′UTR, interestingly, in a let-7 independent manner [78]. Future studies should focus on the other factors and signaling pathways within the stem cell niche under the control of LIN28. The knowledge about mechanisms controlling stem cells’ behavior in the niche will facilitate the development of stem-cell-based therapies. In C. elegans, it was shown that low doses of LIN28 promote longevity but also balance reproduction by regulating germline stem cells number and regulating energy intake [79]. Based on the LIN28 effect on the improved insulin sensitivity and enhanced tissue regeneration determined in mice, LIN28 could affect the aging delay. However, as the overexpression of LIN28 can also contribute to tumorigenesis, lower doses of the protein probably could have a better effect [37,76]. 

## 5. Molecular Pathways of LIN28 Family

### 5.1. Network of LIN28AB Targets

The molecular mechanisms that regulate pluripotency and self-renewal have been extensively investigated in embryonic stem cells (ESCs) that provide insights into early development. The knowledge about such mechanisms is important to improve the potential of pluripotent stem cells for therapeutic applications. On the other hand, germ cells can also provide a novel perspective for the regulation of pluripotent states, mainly because of their potential to reacquire pluripotency via fertilization, teratocarcinogenesis, or spontaneous conversion during culture [80]. A study showed that pluripotency factors LIN28AB mediate the regulation of several RNA-binding proteins (RBPs) and promote their expression [49].

The exact mechanism by which LIN28 affects target mRNA function involves multiple levels of regulation. LIN28 mediates mRNA degradation by recruiting TUTase to uridylate mRNA 3 end [81] or changes the secondary structure of mRNA by recruiting RNA helicase [82]. Moreover, LIN28 also regulates alternative splicing and alternative polyadenylation of target mRNAs [83]. One of the first mRNAs discovered to be bound by LIN28A was the insulin-like growth factor 2 (Igf2) in skeletal myoblasts. LIN28A recruits Igf2 mRNA to polysomes through interactions with translation initiation complexes and enhances Igf2 expression [84]. An in vitro study showed that exogenous LIN28A expression has a proliferative and pro-survival impact on primary cortical neurons by upregulating Igf-2 and inhibiting caspase-3-dependent programed cell death [85]. Recent studies have confirmed the involvement of the LIN28–IGF2 axis in neurogliogenesis. Xia et al. found that LIN28A is an important intrinsic factor that regulates the generation of neurons and glia through IGF-2 signaling [41]. It is known that the Igf pathway involves activation of mitogen-activated protein kinase (MEK) and phosphoinositide-3 kinase (PI3K) by RAS and RAF, as well as activation of the mechanistic target of the rapamycin (mTOR) pathway by AKT [86,87]. Considering the available evidence, it is likely that LIN28 participates in the Igf pathway by protecting Igf-2 and Igf2bp1/2/3 mRNA from degradation [88] or/and enhancing their translation [84,89]. Interestingly, overexpression of LIN28 in injured retinal ganglion cell (RGC) axons can promote their regeneration mediated by Igf1 [90]. LIN28A can stimulate the translation of other mRNA targets, including Cyclin A/B and CDK4, via direct binding to their 3 untranslated region (3 UTR) [91] or the coding region of histone H2 mRNA [92]. In human ES cells (hESCs), LIN28A promotes the translation of reprograming factor OCT4 through binding to the coding region of OCT4 mRNA and interaction with RNA helicase A [93]. Furthermore, a study on human ES and somatic cells found LIN28A preferable to a consensus GGAGA motif enriched within exons and 3′UTR of over 6000 mRNA of genes genome-wide. This study also revealed positive feed-forward autoregulation of LIN28A, directly amplifying its translation [83]. A study on mouse ES cells showed that LIN28A recognizes the mRNA motifs AAGNNG, AAGNG, and, less frequently, UGUG [94]. Other genome-wide studies show that LIN28 associates with mRNA encoding RNPs, metabolic enzymes, and structural constituents of ribosomes, important proteins for cell growth and survival [95]. A very recent study discovered over 12,000 mRNA targets of LIN28A in mouse undifferentiated spermatogonia through high-throughput sequencing of RNAs isolated by crosslinking immunoprecipitation (HITS-CLIP). Further analysis of LIN28A binding sites within mRNA sequences confirmed enrichment in GGAG(A) motifs at the 3′UTR. The same study showed LIN28A’s mechanism of action in maintaining spermatogonial phenotype in the testis by binding to the GGAG(A) motifs of meiotic genes, such as Hormad1, Terb1, and Prdm9, and enhancing their translation during meiosis [96].

Like LIN28A, LIN28B primarily regulates the expression of genes involved in protein translation, mRNA splicing, and cell cycle regulation. A study showed that Lin28b knockdown strongly impaired cell cycle and cell proliferation. Accordingly, some of the genes controlling core signaling pathways directly bound to LIN28B are CDK1, NRAS, RAN, and ERK. Moreover, ribosomal proteins are among the top LIN28B targets and have shown intense changes in their expression following Lin28b knockdown [97]. These findings are consistent with studies that showed that Lin28 mutants have defective phenotypes considering growth and metabolism. Additionally, direct LIN28B-binding targets are HMGA2 and IGF2BP2, which showed a twofold decrease in protein production upon Lin28b knockdown [97]. Since HMGA2 and IGF2BP2 are type-2-diabetes-associated genes, these results are in line with studies showing aberrations in glucose metabolism after the loss of LIN28B [98]. In addition, LIN28B has been shown to bind thousands of other human RNAs directly. Studies showed several possible target motifs for the LIN28B CSD domain. For example, Nam et al. proposed NGNGAYNNN consensus [7], whereas Mayr et al. revealed a GUNNUNN motif [99]. Further, Graf et al. reported AAGRWG (R = A or G) motif [97], which is highly similar to the LIN28A consensus sequence identified by Wilber and colleagues [83].

**Figure 3:**
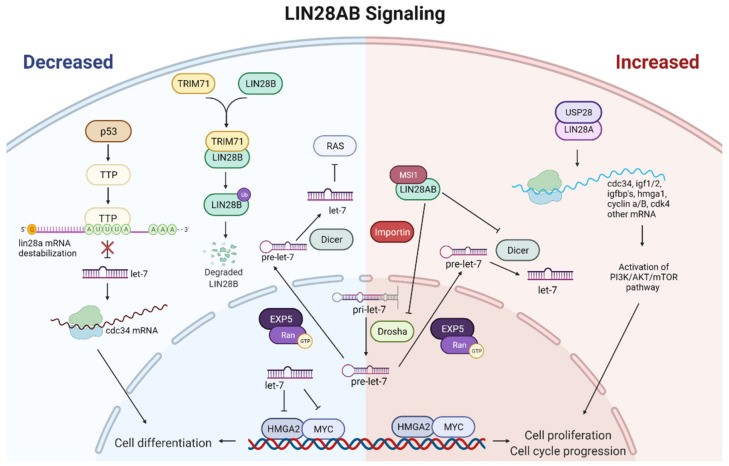
Schematic representation of the main LIN28A and LIN28B signaling. The left panel of the figure shows the cellular effect of decreased LIN28AB. If LIN28A/B are blocked with upstream regulators, biogenesis of let-7 miRNAs proceeded normally. High levels of let-7 will target and reduce the expression of proliferation-associated genes, driving the cells towards differentiation. The right panel of the figure shows the cellular effect of increased LIN28AB. LIN28AB represses the biogenesis of let-7 miRNAs by binding pri-let-7 and pre-let-7 and inhibiting their processing into mature let-7. Due to the low let-7 level, increased expression of proliferation-associated genes can be expected, leading to increased cell proliferation. Red arrows indicate upregulation, and green arrows indicate downregulation. Figure created with BioRender.com (accessed on 4 May 2022).

Molecular mechanisms of LIN28AB action include not only binding to mRNAs, but certain microRNAs as well. The most important developmental partners of LIN28AB are let-7 miRNAs. There are 12 let-7 family members in humans (let-7a-1, -2, -3; let-7b; let-7c; let-7d; let-7e; let-7f-1, -2; let-7g; let-7i; and miR-98) located at eight different chromosomal loci [100]. They act as tumor suppressors by inhibiting the expression of oncogenes, such as MYC, RAS, and HMGA2 [101,102,103] (Figure 3). LIN28A and LIN28B selectively repress let-7 miRNA expression via their RNA-binding domains (RBDs) [48,104]. This LIN28/let-7 axis regulates the normal development and differentiation of ESCs, operating as a switch to maintain an embryonic or differentiated cell fate [105]. Post-transcriptional regulation of let-7 by LIN28 is also required for glucose homeostasis. Studies have shown that LIN28AB in transgenic mice contributes to insulin sensitivity and reduced glucose levels in peripheral blood. In contrast, let-7 overexpression in mice has the opposite effect by promoting higher glucose levels and lower insulin sensitivity [89,106]. Furthermore, many studies have implicated the LIN28/Let-7 pathway in a growing list of numerous cancers [107,108,109], including germ cell tumors [110,111]. These studies demonstrate that low expression of let-7 and high expression of LIN28AB correlates with tumor aggressiveness and poor prognosis [112,113,114,115]. Furthermore, a study on human hepatocellular carcinoma (HCC) shows that aberrant expression of LIN28AB and let-7 facilitates aerobic glycolysis or the Warburg effect. They found that LIN28AB enhances, while let-7 suppresses, aerobic glycolysis by targeting pyruvate dehydrogenase kinase 1 (PDK1), demonstrating a novel pathway to mediate aerobic glycolysis of cancer cells [116]. In accordance with a previous study, Yuko et al. showed that LIN28A therapeutic effect on CTSCs was linked to the let-7/PDK1 signaling pathway [117]. Insight into the exact mechanisms of the LIN28/let-7 axis could enable the manipulation of cellular pluripotency and diseases such as diabetes and cancer. Indeed, significant efforts have been made to elucidate the mechanism underlying LIN28AB mediated let-7 suppression. A double-negative feedback loop between LIN28AB and let-7 where LIN28AB binds to either pri-let-7 or pre-let-7 via its CSD and CCHC domains was reported [118]. CCHC domains dimerize on a GGAG motif adjacent to the Dicer cleavage site and, thus, prevent the processing of let-7 precursor by Dicer and Drosha, while CSD inserts into the apical part of the precursor loop [7,119,120] (Figure 3). Consequently, LIN28AB causes oligo-uridylation at the 3 terminal of pre-let-7 recruiting uridylyltransferase (TUTase) [121,122]. In humans, only TUT4 (ZCCHC11) and TUT7 (ZCCHC6) were shown to actively oligo-uridylate pre-let-7 [123]. A study demonstrated that CSD binds to let-7 with higher affinity than the ZKD, and that CSD alone has fast-on and fast-off binding kinetics [99,124]. However, the interaction between the ZKD and the GGAG sequence, despite its low binding affinity, increases the half-life of the LIN28A/pre-let-7 complex and stabilizes it. The LIN28/let-7 complex stabilization prevents dissociation and is crucial for TUT4 recruitment [124]. Thus, LIN28A/LIN28B inhibits let-7 biogenesis and induces its degradation (Figure 3). On the other hand, let-7 may inhibit the expression and function of LIN28A/LIN28B binding to 3UTR of their mRNAs [125]. Recently, a novel -(U)GAU- motif was identified on pre-let-7, similar to the CSD-binding consensus sequence, that promotes stronger binding of LIN28 to the pre-let-7 subclass containing (U)GAU (CSD+) than to the pre-let-7 subclass without this motif. Furthermore, the in vivo uridylation of CSD- let-7 members by LIN28 was less efficient than the uridylation of the CSD+ let-7 subclass [126]. Sequence and structural analysis revealed functional equivalency between mouse LIN28A and human LIN28 paralogs in the recruitment of TUTase. Wang et al. found an LIN28-binding sequence (a YRYFACPQKK motif) that is conserved between TUT4 and TUT7 and between TUT4 in mice and humans [124]. Altogether, this wide network of LIN28 targets allows it to program cell metabolism, growth, and self-renewal. It will be interesting to investigate further the interplay between LIN28 and TUTase and whether miRNAs other than let-7 are substrates for TUT4/TUT7. 

### 5.2. Upstream Regulators of LIN28AB 

Reactivation of LIN28AB in disorders such as GCNIS can be triggered by epigenetic changes and upstream modulators and/or loss of transcriptional repressors. However, much remains to be clarified about the exact mechanisms which lead to LIN28AB aberrant expression. The mechanism of DNA methylation on gene promoters containing CpG islands represents one layer of epigenetic regulation essential for controlling gene expression. Novel research found that LIN28A expression can be silenced via CpG island hypermethylation. Xu et al. reported that LIN28A expression is directly associated with the methylation status of promoter-associated CpG islands in pancreatic cancer cells. The first CpG island was identified in the first exon and the second was in the first intron [127]. Since LIN28A is overexpressed in GCNIS, it would be worthwhile to investigate the association of its expression with the methylation status of these CpG islands. Regarding LIN28B paralog, a recent study found that its expression could be activated by hypomethylation of four specific CpG in the LIN28B promoter [128]. Together, these results suggest that DNA methylation of specific CpG sites in LIN28AB promoter could be a potential molecular marker for prognosis prediction and individualized treatment among patients with different tumors. It would be worth investigating the methylation status of these CpG sites of the LIN28AB promoter in patients with testicular tumors.

Post-translational modifications of LIN28A and LIN28B, such as methylation, acetylation, phosphorylation, and ubiquitination and their effects on LIN28AB protein stability and activity, have not yet been fully characterized. However, it is known that the balance of post-translational modifications, such as acetylation and deacetylation, essentially affect LIN28 protein regulation. For example, one of the known acetyltransferases, P300/CBP-associated factor (PCAF), directly acetylates LIN28 and causes the reduction in its level. On the other hand, deacetylase SIRT1 reverses this process [129]. A recent study showed that MAPK/ERK-mediated phosphorylation also regulates the function of LIN28 as a pluripotency factor [130]. One of the deubiquitinating enzymes (DUBs), ubiquitin-specific protease 28 (USP28), is reported to have a critical regulatory effect on the LIN28A by deubiquitinating the protein. Furthermore, USP28 stabilizes LIN28A and extends its half-life by antagonizing LIN28A protein turnover and reversing its proteasomal degradation. Moreover, USP28 increases the LIN28-mediated inhibition of let-7 (Figure 3). Ultimately, USP28 induces the tumorigenic function of LIN28A and enhances the viability and migration of cancer cells, increasing LIN28A-mediated tumor progression [131]. These data drive the synergistic, combinatorial approach of targeting LIN28A and USP28 in contributing to the effectiveness of cancer therapeutics. Another LIN28A upstream regulator is tristetraprolin (TTP), an AU-rich element (ARE) binding protein. Contrary to USP28, TTP binds to the first AUUUA pentamer (ARE1) within the Lin28 mRNA 3′-UTR, destabilizing it, and, therefore, downregulates the expression of Lin28. TTP-induced downregulation of Lin28 increases the expression of let-7b miRNA, which blocks the translation of CDC34, an mRNA involved in cell cycle progression. This is a likely mechanism by which TTP-mediated Lin28a repression inhibits the growth of human cancer cells [132]. Moreover, TTP represents a significant link between p53 activation caused by the DNA damage and the Lin28/let-7 axis. Lee et al. demonstrated that p53 stimulates TTP expression in cancer cells after treatment with doxorubicin (DOX), a DNA-damaging agent. TTP, in turn, increased the level of let-7 by repressing Lin28a (Figure 3). In accordance with the above results, cancer cells with p53 mutations failed to induce the let-7 expression after DOX treatment. Likewise, inhibition of TTP by siRNAs attenuated the inhibitory effect of DOX on let-7 expression and cancer cell growth. Further studies on the p53-TTP-Lin28-let-7 system can help understand the occurrence of chemoresistance in human cancers [133]. In addition, p53 can influence Lin28b paralog as well. A recent study showed that the premature onset of puberty in mice fed with a high-fat diet (HFD) is controlled by p53-c-Myc/Lin28b/let-7 pathway. In the high-fat diet mice, c-Myc and Lin28b levels increased, while let-7a mRNA expression decreased. Overexpression of p53 in the hypothalamus of HFD mice reduced c-Myc and Lin28b on both mRNA and protein levels while concomitantly increasing the expression of let-7a mRNA. In contrast, inhibition of p53 increased c-Myc and Lin28b expression but reduced let-7a levels. In this way, high fat intake can accelerate puberty onset by upregulating p53 expression, which accelerates hypothalamic–pituitary–gonadal axis activation through the c-Myc/Lin28/let-7 system [134].

One of the most studied upstream post-transcriptional modulators of the LIN28B paralog is tripartite motif-containing 71 (TRIM71), also known as lineage variant 41 (lin-41). TRIM71 is a member of the TRIM-NHL family, together with TRIM2, TRIM3, and TRIM32 [135]. All TRIM-NHL members share structural similarities and possess functional E3 ubiquitin ligase activity because of the RING domain in the N-terminus [136]. The study showed that TRIM71 negatively regulates protein stability of LIN28B, but not LIN28A, by catalyzing polyubiquitination. Further, a C-terminal unique amino acid stretch of LIN28B and N-terminal RING finger motif of TRIM71 are essential for protein–protein interaction and polyubiquitination and consequent proteasomal degradation of LIN28B. Moreover, TRIM-71-mediated downregulation of LIN28B led to increases in the let-7 expression and repression of one of the known let-7 targets, HMGA2 [137] (Figure 3). Interestingly, TRIM71 expression is downregulated in various cancer tissues in which the LIN28B–let-7–HMGA2 signaling pathway is conserved compared with normal tissue counterparts. Furthermore, the specific knockdown of TRIM71 increased the proliferation of cancer cells. In contrast, overexpression of TRIM71 in non-small cell lung carcinoma (NSCLC) cells, in which the LIN28B–let-7–HMGA2 pathway was conserved, decreased cancer cell phenotypes [138]. Altogether, these data suggest that the oncogenic activity of LIN28B is repressed post-transcriptionally by TRIM71. A recent study showed that TRIM71 was differentially expressed among good- and poor-quality bull semen samples [139]. Additional studies are needed to investigate the potential role of TRIM71 and the other TRIM-NHL members as upstream regulators of the LIN28B–let-7 axis in testis, especially in humans. Except by proteins, regulation of LIN28AB expression can be achieved by microRNAs. Aside from let-7 [125], other microRNAs can inhibit the expression and function of LIN28AB. For example, the miR-30 family, miR-9 [140], miR-125 [140,141], and miR-181 [142] have been reported to downregulate LIN28 in ESCs and cancers cells. Interestingly, these microRNAs are under-expressed in malignant germ cell tumor (GCT) [143]. Figure 3 shows a schematic representation of the main LIN28AB signaling pathways involved in controlling cell renewal, maturation, fertility, aging, and tumor development.

## 6. LIN28AB in Testicular Disorders

As mentioned previously, we can observe high expression of LIN28A and low let-7 in numerous cancers [144], including germ cell tumors [22,143,145], implying that LIN28/let-7 is a common regulatory circuit shared by stem cells and cancer cells. By loss-of-function analysis, a study on mouse experimental teratoma models revealed the function of LIN28 in the genesis of mammalian germ cell tumors. Furthermore, LIN28A as well as its homolog LIN28B are consistently expressed in malignant germ cell tumors, including yolk sac tumors and choriocarcinomas [111]. In accordance with that, Cao et al. defined LIN28 as a highly sensitive marker for testicular germ cell tumors, including GCNIS, classic seminomas, embryonal carcinomas, and yolk sac tumors [110]. Together, these data implicate LIN28 as a diagnostic marker of germ cell malignancy. Further investigations are required to establish the clinical significance of LIN28A as an ideal prognostic biomarker in human testicular cancers. 

Recent works suggest a role of LIN28/let-7pathway in the control of spermatogenesis and fertility. A study on Lin28 knockout (KO) mice found that Lin28a deficiency reduces the number of germ cells during embryogenesis, leading to impaired fertility in adults. Even though Lin28 can affects protein translation independently of let-7, the study showed that overexpression of let-7 results in the same phenotype as Lin28a deficiency, which confirms a regulative role of the LIN28/let-7 axis in establishing germ cell pools during embryogenesis [70]. Further, germ-cell-specific Lin28a KO mice have been shown to reduce testis weight and sperm number and impair the proliferation of spermatogonial cells [146]. However, limited information regarding its expression in human infertile testis requires further research to analyze the correlation between the human LIN28AB/let-7 axis with infertility. 

A study by Werler et al. has shown decreased LIN28 expression in a mouse model for Klinefelter’s syndrome (KS). Unlike the control group of mice, where LIN28 was found in gonocytes and spermatogonia in the first 10 days postpartum (dpp), in the KS model, LIN28 was only expressed in a few gonocytes on day one postpartum (pp) and some spermatogonia up till day 3pp. However, no LIN28 expression was observed from day 5pp onwards. In the further stages, at day 14 and 21pp, two KS animals had LIN28 signal in the spermatogenic foci in some tubules with ongoing spermatogenesis, while surrounding tubules lacking germ cells did not show any expression [147]. Analysis of mRNA Lin28 confirmed its presence from day 1pp onwards in controls with increasing levels during postnatal development up to 14pp. In contrast, it was decreased later in adulthood, which is consistent with findings of Gaytan, Francisco, et al. and Zheng, Ke et al. [72,148]. In contrast, only weak Lin28 mRNA expression was detected in KS mice from day 1 to 14dpp, and a complete absence of Lin28 was observed in adult animals [147]. 

LIN28AB expression patterns can be disturbed by the lack of GPR54 expression, a gene controlling the onset of puberty. A study on a mouse model of hypogonadotropic hypogonadism (Gpr54 KO) showed a lack of LIN28B in testis, while LIN28A-positive cells were present but with reduced total testicular expression. Moreover, LIN28A in Gpr54 KO mice showed irregular distribution through the testis. In addition, Gpr54-null mice showed enhanced levels of let-7a/b in contrast to wild-type (WT) mice. Adult Gpr54 KO mice showed small seminiferous tubules with the most advanced germ cells corresponding to leptotene spermatocytes and the lack of differentiated Leydig cells, which may explain the absence of LIN28B. However, gonadotropin administration (hCG and FSH) to Gpr54-null mice can rescue such defective expression [72]. Further, analyses of the testicular LIN28AB/let-7 axis in rat models of perturbed puberty revealed that early manipulation of the hormonal and nutritional environment and photoperiod manipulation could influence the expression levels of the LIN28AB and let-7, along with postnatal testicular maturation [4]. Together, these results emphasize the role of GH in testicular function and the putative involvement of changes in the LIN28/let-7 axis. Further research is needed to elucidate the potential role of both LIN28A and LIN28B in the feedback of steroid hormones in the testis to GnRH and/or FSH/LH. 

Interestingly, recent studies on mice have shown aberrant LIN28AB expression in the testis after prenatal exposure to endocrine-disrupting chemicals (EDC) [149,150]. As previous studies have shown, testicular exposure to ED chemicals in prenatal development may impair PGC differentiation, causing decreased sperm quality [151] and number and increased apoptosis of germ cells [152], as well as infertility in adults [153]. Accordingly, fetal and neonatal exposure to ED chemicals bisphenol-A and stigmasterol (BS) alters adult germ cell physiology. It induces testis histology defects as well as fertility disorders, associated with altered spermatogenesis in adult mice at 3 or 6 months of age. Interestingly, in vitro exposure of spermatogonial cell line to BS decreased LIN28 level [149]. Another study on mice showed that embryonic exposure to vinclozolin (VCZ), a widely used fungicide with antiandrogenic effects, deregulates Lin28a/Lin28b/let-7 pathway in three successive generations of males. More precisely, exposure to VCZ decreased Lin28a in PGCs from F1 and F2 males, whereas its level was slightly increased in F3, similar to Lin28b decreased in F1/F2 and normalized in F3 PGCs. Furthermore, decreased levels of Lin28ab correlate with upregulation of the precursor and some mature forms of let-7 [150]. Altogether, these results demonstrate that embryonic exposure to environmental EDC can cause transgenerational effects on germ cells through Lin28a/Lin28b/let-7 pathway.

## 7. The LIN28AB as a Molecular Target in Treating Injuries and Diseases

First of all, LIN28 has been researched as a target in tumor therapy. Unlike well-differentiated cancer cells, cancer stem cells (CSCs) present in many undifferentiated tumors seem to be more sensitive to chemotherapy and radiation therapy. An example of an undifferentiated tumor is GCNIS, a pathological precursor lesion for testicular germ cell tumors (TGCT) in adolescents and adult men [65,154]. Since the dysregulation of the LIN28/let-7 axis is present in malignant GCTs, this pathway suggests a promising target for developing novel therapeutic agents [145]. Many other tumors have already found a correlation between the LIN28AB/let-7 axis and anticancer treatment. Studies have shown that downregulation of LIN28A in chemotherapy and radiation-resistant breast cancer cells enhanced their sensitivity to the treatments [155,156]. In another study, LIN28AB/let-7 axis was involved in the resistance of human pancreatic and lung cancer cells [157]. Furthermore, in vitro experiments on human glioblastoma cells showed that suppression of LIN28 by small hairpin RNA (shRNA) caused cell cycle arrest in the G1 phase, delayed cell proliferation, increased apoptosis, and resulted in fewer colonies compared to controls [158]. A study showed that downregulation of LIN28B reduced self-renewal ability and increased let-7 level in the prostate cancer cells [159]. Another study demonstrated that knockdown of Lin28 decreased viability and promoted apoptosis in colorectal cancer cells, whereas this effect was attenuated by let-7c inhibition [160]. A recent study found that treatment with an LIN28 inhibitor C1632 increases let-7 and suppresses programmed death ligand 1 (PD-L1) expression, leading to reactivation of antitumor immunity in vitro and in vivo. In addition, C1632 also displayed the capacity to inhibit cancer cell proliferation and tumor growth in mice [161]. When looking at the LIN28-regulated oncogenes, analyses of CRISPR-engineered cells suggest that the LIN28/let-7 axis regulates MYC and cell cycle pathways in multiple myeloma and provides proof of principle for therapeutic regulation of MYC through let-7. This study demonstrated that high levels of let-7 expression repress tumor growth by regulating MYC expression [114]. These findings reveal a mechanism of therapeutic targeting of MYC through the LIN28B/let-7 axis that may impact other reproductive MYC-dependent cancers, such as testicular cancer [162]. Therefore, inhibition of LIN28 has a dual effect on cancer therapy: stimulating antitumor immune responses and suppressing tumor growth by inhibiting cancer cell proliferation. Notably, Roos et al. identified that N-methyl-N-[3-(3-methyl[1,2,4]triazolo[4,3-b]pyridazin-6-yl)phenyl]acetamide, which blocks the Lin28/let-7 interaction, rescued let-7 processing and function, and induced differentiation of mouse ESCs, eventually reducing tumor-sphere formation in human prostate and hepatocellular carcinoma cells [163]. These findings may represent a new direction for treating germ cell tumors. More studies must be performed to explore small-molecule inhibitors of LIN28 further and evaluate their therapeutic potential in germ cell tumor pathologies. L. Wang et al. suggest that a potentially druggable pocket locates between the two zinc knuckles (ZKD) within LIN28 [124]. Further, the same group developed a high-throughput screening strategy to identify small-molecule inhibitors for both CSD and ZKD domains of LIN28 involved in let-7 interactions. Using fluorescence polarization assay, they found that the LIN28 inhibitor LI71 binds the CSD to suppress LIN28′s activity in leukemia cells and embryonic stem cells, while another inhibitor TPEN destabilizes the ZKD of LIN28 [164]. In vitro and in vivo studies on oral squamous cell carcinoma (OSCC) showed the synergistic antitumor effect of an LIN28 inhibitor C1632 and hypoglycemic medication with metformin. C1632 inhibits LIN28 and thereby regulates LIN28/let-7 axis and, together with metformin, reduces proliferation, migration, and self-renewal capacities of OSCC cells [165]. These results demonstrate selective pharmacologic inhibition of individual domains of LIN28 and provide a foundation for therapeutic inhibition of the let-7 biogenesis pathway in LIN28-driven diseases. Therefore, novel therapeutic agents designed to target the LIN28AB/let-7 axis might help achieve better therapeutic outcomes for the treatment of chemotherapy and radiation-resistant tumors, such as testicular cancer.

Except in cancer treatment, LIN28 can be a target for other pathological conditions, e.g., injuries and diseases resulting from tissue damage, due to its positive effect on cell renewal. For example, induction of LIN28A to transgenic mice promotes glucose tolerance and tissue repair by enhancing oxidative metabolism needed to activate adult cells out of quiescence [38]. Another study showed that reintroduction of LIN28a to adult CTSC (CTSC-LIN) identifies a novel role for LIN28a in cardiac regeneration after ischemic injury. LIN28A promotes energetic activity by increasing oxidative phosphorylation and glycolysis in adult CTSCs. Furthermore, CTSC-LIN had a significantly increased amount of antioxidant markers and reduced ROS generation compared to the control (CTSC-GFP). In addition, CSTC-LIN showed an increased ability to secrete pro-reparative paracrine factors under hypoxic conditions and promote cardiomyocyte proliferation and survival after ischemic cardiac injury [117]. In other words, LIN28 makes adult cardiac stem cells more metabolically flexible, significantly improving their chances of survival. Furthermore, LIN28A enhances the therapeutic potential of cultured neural stem cells in a Parkinson’s disease model [166] and promotes axonal regeneration after optic nerve and spinal cord injury in mice [167]. Based on LIN28-mediated repair enhancement in multiple tissues and the main pieces of information obtained in these studies, targeting LIN28 can be applied to stem-cell-based therapeutic approaches in some reproductive hormonal-related disorders, such as hypogonadotropic hypogonadism or chemotherapy-induced testicular injury.

## 8. Conclusions and Future Prospects

There is a spatial and temporal restriction of the differentiation potential of originally pluripotent germ cells during the testis development. Differentiation of a particular cell type depends on qualitative and quantitative differences in the expression of a specific set of genes. Studies have shown that perturbed expression of LIN28AB during the fetal and early postnatal periods may induce phenotypic changes in adults [36,37,72,77,145]. Therefore, it is important to determine time windows during early development, which are critical for phenotypic changes in adulthood. The first window is in the early fetal period, where the gene’s actions closer to the start of masculinization programing might have a stronger impact on puberty and growth, while the second window comes later during the juvenile development (Figure 1 and Figure 2). Based on previous findings, two paralogs of LIN28 have a partly overlapping but also different role in human testis development. While the testicular role of LIN28A in germline stem cells’ self-renewal is well established, the role of its paralog LIN28B in the testis has not been elucidated (Table 1). There are only a few studies about LIN28B expression in Leydig cells and spermatids [4,23,72], but no one has yet described its role there. Blocking their expression in a specific time window, e.g., at 4 WGA or in the middle of the infantile period, where they showed the highest expression, could further clarify their role in the testis. It will be very interesting to investigate the possible endocrinological roles of LIN28AB in the testis. To achieve that, future research should be based on expanding knowledge about the already known LIN28AB pathways in the testis, such as the LIN28AB–let-7 axis [4,72]. In addition, the LIN28AB pathways and molecular partners detected in other tissues should also be considered in explaining the testicular network (Figure 3). As some studies showed, impaired LIN28AB pathway can lead to several testicular disorders, among which are testicular germ cell tumors [22,110,111,140], hypogonadotropic hypogonadism [72], Klinefelter’s syndrome [147], and other types of infertility [69,141,145,146]. There is growing evidence that targeting LIN28AB can be applied to stem-cell-based therapeutic approaches in various disorders [114,155,156,157,158,159,160,161,162,163,164,165] or tissue injuries [38,117,167], indicating a possibility of targeting these paralogs in testicular disorder as well.

Overall, the accumulating data suggest that LIN28AB has the potential to affect testis development and fertility, as well as pubertal timing, through separate mechanisms and acting at different times during development. LIN28A and LIN28B might present potential targets for developing new therapies in the treatment of fertility but also testicular tumors.

## Figures and Tables

**Table 1:** The role of LIN28AB in various cells and tissues.

LIN28	Role	Model	Reference
**LIN28AB**	stem cell self-renewal ^1^ cell reprograming ^2^	mouse ^1,2^ and human ^1^	[24] ^1^ [25] ^1,2^
	ESCs differentiation	human	[8,9]
	body size ^1^ onset of puberty ^2^	transgenic mouse ^1,2^ human ^2^	[33] ^1,2^ [35] ^1,2^ [37] ^1,2^ [34] ^2^ [36] ^2^
	tail development	mouse	[39]
	tooth development	mouse	[40]
	trophoblast proliferation	human ^1^ and sheep ^2^	[16] ^1^ [43] ^1^ [45] ^2^
	developmental timing	C. elegans	[5]
**LIN28A** **paralog**	heart development	mouse	[12]
	neurogliogenesis	Rat and mouse	[41]
	ovulation	C. elegans	[42]
	trophoblast differentiation	human	[15]
	mesodermal and neural cell fate	mouse	[39]
	hippocampal neurogenesis	mouse	[11]
	germline stem cells self-renewal	human	[22] ^1^ [23] ^1^
	body height	human	[28,29,30]
**LIN28B** **paralog**	finger length ratio	human	[31]
	adiposity	human	[32]
	placenta development	human	[43,44]
	hematopoietic maturation	mouse	[13];
	age at menarche	human	[33]

^1^ and ^2^ connect the role to appropriate model and reference numbers.

## Data Availability

Not applicable.

## Abbreviations

CysCysHisCys	CCHC
CSD	cold-shock domain
CSP	cold shock proteins
CTSC	cardiac tissue-derived stem-like cell
DOX	doxorubicin
dpc	days post coitum
dpp	days postpartum
EDC	endocrine-disrupting chemicals
ESC	embryonic stem cell
GCNIS	germ cell neoplasia in situ
GCT	germ cell tumor
GOF	gain-of-function
hESC	human embryonic stem cell
HFD	high-fat diet
HSPC	hematopoietic stem and progenitor cell
IHC	immunohistochemical
IMP	importin
KS	Klinefelter’s syndrome
LOF	loss-of-function
MPW	masculinization programming window
NLS	nuclear localization signal
NoLS	nucleolar localization signal
OSCC	oral squamous cell carcinoma
PGC	primordial germ cell
preSPG	pre-spermatogonia
PSC	pluripotent stem cell
RPC	retinal progenitor cell
scRNA-seq	single-cell RNA sequencing
SPG	spermatogonia
SSC	spermatogonial stem cell
TGCT	testicular germ cell tumors
WG	weeks of gestation
WGA	weeks of gestational age
ZKD	zinc knuckles domain

## References

[1] Skakkebaek N.E., Rajpert-De Meyts E., Buck Louis G.M., Toppari J., Andersson A.M., Eisenberg M.L., Jensen T.K., Jørgensen N., Swan S.H., Sapra K.J. (2015). Male Reproductive Disorders and Fertility Trends: Influences of Environment and Genetic Susceptibility. Physiol. Rev..

[2] Rotgers E., Jørgensen A., Yao H.H.C. (2018). At the Crossroads of Fate-Somatic Cell Lineage Specification in the Fetal Gonad. Endocr. Rev..

[3] Mäkelä J.A., Koskenniemi J.J., Virtanen H.E., Toppari J. (2019). Testis Development. Endocr. Rev..

[4] Sangiao-Alvarellos S., Manfredi-Lozano M., Ruiz-Pino F., León S., Morales C., Cordido F., Gaytán F., Pinilla L., Tena-Sempere M. (2015). Testicular Expression of the Lin28/Let-7 System: Hormonal Regulation and Changes during Postnatal Maturation and after Manipulations of Puberty. Sci. Rep..

[5] Moss E., Lee R., Ambros V. (1997). Control of Developmental Timing by the Cold Shock Domain Protein Lin-28 and Its Regulation by the Lin-4 RNA. Cell.

[6] Guo Y., Chen Y., Ito H., Watanabe A., Ge X., Kodama T., Aburatani H. (2006). Identification and Characterization of Lin-28 Homolog B (LIN28B) in Human Hepatocellular Carcinoma. Gene.

[7] Nam Y., Chen C., Gregory R.I., Chou J.J., Sliz P. (2011). Molecular Basis for Interaction of Let-7 MicroRNAs with Lin28. Cell.

[8] Darr H., Benvenisty N. (2009). Genetic Analysis of the Role of the Reprogramming Gene LIN-28 in Human Embryonic Stem Cells. Stem. Cells.

[9] Rahkonen N., Stubb A., Malonzo M., Edelman S., Emani M.R., Närvä E., Lähdesmäki H., Ruohola-Baker H., Lahesmaa R., Lund R. (2016). Mature Let-7 MiRNAs Fine Tune Expression of LIN28B in Pluripotent Human Embryonic Stem Cells. Stem. Cell Res..

[10] Viswanathan S.R., Daley G.Q. (2010). Lin28: A MicroRNA Regulator with a Macro Role. Cell.

[11] Hu Z., Ma J., Yue H., Li X., Wang C., Wang L., Sun B., Chen Z., Wang L., Gu Y. (2022). A Role for Lin28a in Aging-Associated Decline of Adult Hippocampal Neurogenesis. bioRxiv.

[12] Kurian J., Yuko A.E., Kasatkin N., Rigaud V.O.C., Busch K., Harlamova D., Wagner M., Recchia F.A., Wang H., Mohsin S. (2021). Uncoupling Protein 2-Mediated Metabolic Adaptations Define Cardiac Cell Function in the Heart during Transition from Young to Old Age. Stem. Cells Transl. Med..

[13] Yuan J., Nguyen C.K., Liu X., Kanellopoulou C., Muljo S.A. (2012). Lin28b Reprograms Adult Bone Marrow Hematopoietic Progenitors to Mediate Fetal-like Lymphopoiesis. Science.

[14] Vogt E.J., Meglicki M., Hartung K.I., Borsuk E., Behr R. (2012). Importance of the Pluripotency Factor LIN28 in the Mammalian Nucleolus during Early Embryonic Development. Development.

[15] Seabrook J.L., Cantlon J.D., Cooney A.J., McWhorter E.E., Fromme B.A., Bouma G.J., Anthony R.V., Winger Q.A. (2013). Role of LIN28A in Mouse and Human Trophoblast Cell Differentiation. Biol. Reprod..

[16] Ali A., Anthony R.V., Bouma G.J., Winger Q.A. (2019). LIN28-Let-7 Axis Regulates Genes in Immortalized Human Trophoblast Cells by Targeting the ARID3B-Complex. FASEB J..

[17] Canfield J., Arlier S., Mong E.F., Lockhart J., VanWye J., Guzeloglu-Kayisli O., Schatz F., Magness R.R., Lockwood C.J., Tsibris J.C.M. (2019). Decreased LIN28B in Preeclampsia Impairs Human Trophoblast Differentiation and Migration. FASEB J..

[18] Sangiao-Alvarellos S., Manfredi-Lozano M., Ruiz-Pino F., Navarro V.M., Sánchez-Garrido M.A., Leon S., Dieguez C., Cordido F., Matagne V., Dissen G.A. (2013). Changes in Hypothalamic Expression of the Lin28/Let-7 System and Related MicroRNAs during Postnatal Maturation and after Experimental Manipulations of Puberty. Endocrinology.

[19] Barbaux S., Gascoin-Lachambre G., Buffat C., Monnier P., Mondon F., Tonanny M.B., Pinard A., Auer J., Bessières B., Barlier A. (2012). Genome-Wide Approach Reveals Novel Imprinted Genes Expressed in the Human Placenta. Epigenetics.

[20] Chan H.W., Lappas M., Yee S.W., Vaswani K., Mitchell M.D., Rice G.E. (2013). The Expression of the Let-7 MiRNAs and Lin28 Signalling Pathway in Human Term Gestational Tissues. Placenta.

[21] Ali A., Bouma G.J., Anthony R.V., Winger Q.A. (2020). The Role of LIN28-Let-7-ARID3B Pathway in Placental Development. Int. J. Mol. Sci..

[22] Gillis A.J.M., Stoop H., Biermann K., van Gurp R.J.H.L.M., Swartzman E., Cribbes S., Ferlinz A., Shannon M., Oosterhuis J.W., Looijenga L.H.J. (2011). Expression and Interdependencies of Pluripotency Factors LIN28, OCT3/4, NANOG and SOX2 in Human Testicular Germ Cells and Tumours of the Testis. Int. J. Androl..

[23] Aeckerle N., Eildermann K., Drummer C., Ehmcke J., Schweyer S., Lerchl A., Bergmann M., Kliesch S., Gromoll J., Schlatt S. (2012). The Pluripotency Factor LIN28 in Monkey and Human Testes: A Marker for Spermatogonial Stem Cells?. Mol. Hum. Reprod..

[24] Yu J., Vodyanik M.A., Smuga-Otto K., Antosiewicz-Bourget J., Frane J.L., Tian S., Nie J., Jonsdottir G.A., Ruotti V., Stewart R. (2007). Induced Pluripotent Stem Cell Lines Derived from Human Somatic Cells. Science.

[25] Zhang J., Ratanasirintrawoot S., Chandrasekaran S., Wu Z., Ficarro S.B., Yu C., Ross C.A., Cacchiarelli D., Xia Q., Seligson M. (2016). LIN28 Regulates Stem Cell Metabolism and Conversion to Primed Pluripotency. Cell Stem. Cell.

[26] Zhou W., Choi M., Margineantu D., Margaretha L., Hesson J., Cavanaugh C., Blau C.A., Horwitz M.S., Hockenbery D., Ware C. (2012). HIF1α Induced Switch from Bivalent to Exclusively Glycolytic Metabolism during ESC-to-EpiSC HESC Transition. EMBO J..

[27] Folmes C.D.L., Nelson T.J., Martinez-Fernandez A., Arrell D.K., Lindor J.Z., Dzeja P.P., Ikeda Y., Perez-Terzic C., Terzic A. (2011). Somatic Oxidative Bioenergetics Transitions into Pluripotency-Dependent Glycolysis to Facilitate Nuclear Reprogramming. Cell Metab..

[28] Widén E., Ripatti S., Cousminer D.L., Surakka I., Lappalainen T., Järvelin M.R., Eriksson J.G., Raitakari O., Salomaa V., Sovio U. (2010). Distinct Variants at LIN28B Influence Growth in Height from Birth to Adulthood. Am. J. Hum. Genet..

[29] Lanktree M.B., Guo Y., Murtaza M., Glessner J.T., Bailey S.D., Onland-Moret N.C., Lettre G., Ongen H., Rajagopalan R., Johnson T. (2011). Meta-Analysis of Dense Genecentric Association Studies Reveals Common and Uncommon Variants Associated with Height. Am. J. Hum. Genet..

[30] Lettre G., Jackson A.U., Gieger C., Schumacher F.R., Berndt S.I., Sanna S., Eyheramendy S., Voight B.F., Butler J.L., Guiducci C. (2008). Identification of Ten Loci Associated with Height Highlights New Biological Pathways in Human Growth. Nat. Genet..

[31] Medland S.E., Zayats T., Glaser B., Nyholt D.R., Gordon S.D., Wright M.J., Montgomery G.W., Campbell M.J., Henders A.K., Timpson N.J. (2010). A Variant in LIN28B Is Associated with 2D:4D Finger-Length Ratio, a Putative Retrospective Biomarker of Prenatal Testosterone Exposure. Am. J. Hum. Genet..

[32] Leinonen J.T., Surakka I., Havulinna A.S., Kettunen J., Luoto R., Salomaa V., Widén E. (2012). Association of LIN28B with Adult Adiposity-Related Traits in Females. PLoS ONE.

[33] He C., Kraft P., Chen C., Buring J.E., Paré G., Hankinson S.E., Chanock S.J., Ridker P.M., Hunter D.J., Chasman D.I. (2009). Genome-Wide Association Studies Identify Loci Associated with Age at Menarche and Age at Natural Menopause. Nat. Genet..

[34] Ong K.K., Elks C.E., Li S., Zhao J.H., Luan J., Andersen L.B., Bingham S.A., Brage S., Smith G.D., Ekelund U. (2009). Genetic Variation in LIN28B Is Associated with the Timing of Puberty. Nat. Genet..

[35] Perry J.R.B., Stolk L., Franceschini N., Lunetta K.L., Zhai G., McArdle P.F., Smith A.V., Aspelund T., Bandinelli S., Boerwinkle E. (2009). Meta-Analysis of Genome-Wide Association Data Identifies Two Loci Influencing Age at Menarche. Nat. Genet..

[36] Leinonen J.T., Chen Y.C., Tukiainen T., Panula P., Widén E. (2019). Transient Modification of Lin28b Expression—Permanent Effects on Zebrafish Growth. Mol. Cell. Endocrinol..

[37] Zhu H., Shah S., Shyh-Chang N., Shinoda G., Einhorn W.S., Viswanathan S.R., Takeuchi A., Grasemann C., Rinn J.L., Lopez M.F. (2010). Lin28a Transgenic Mice Manifest Size and Puberty Phenotypes Identified in Human Genetic Association Studies. Nat. Genet..

[38] Shyh-Chang N., Zhu H., Yvanka De Soysa T., Shinoda G., Seligson M.T., Tsanov K.M., Nguyen L., Asara J.M., Cantley L.C., Daley G.Q. (2013). XLin28 Enhances Tissue Repair by Reprogramming Cellular Metabolism. Cell.

[39] Robinton D.A., Chal J., Lummertz da Rocha E., Han A., Yermalovich A.V., Oginuma M., Schlaeger T.M., Sousa P., Rodriguez A., Urbach A. (2019). The Lin28/Let-7 Pathway Regulates the Mammalian Caudal Body Axis Elongation Program. Dev. Cell.

[40] Dong N., Liu Y., Zhang T., Zhao L., Tian J., Ruan J. (2017). Different Expression Patterns of Lin28 and Lin28b in Mouse Molar Development. Arch. Oral Biol..

[41] Xia X., Teotia P., Ahmad I. (2018). Lin28a Regulates Neurogliogenesis in Mammalian Retina through the Igf Signaling. Dev. Biol..

[42] Choi S., Ambros V. (2019). The C. Elegans Heterochronic Gene Lin-28 Coordinates the Timing of Hypodermal and Somatic Gonadal Programs for Hermaphrodite Reproductive System Morphogenesis. Development.

[43] West R.C., McWhorter E.S., Ali A., Goetzman L.N., Russ J.E., Gonzalez-Berrios C.L., Anthony R.V., Bouma G.J., Winger Q.A. (2019). HMGA2 Is Regulated by LIN28 and BRCA1 in Human Placental Cells. Biol. Reprod..

[44] Santoro G., Lapucci C., Giannoccaro M., Caporilli S., Rusin M., Seidenari A., Ferrari M., Farina A. (2021). Diagnostics Abnormal Circulating Maternal MiRNA Expression Is Associated with a Low (<4%) Cell-Free DNA Fetal Fraction. Diagnostics.

[45] Ali A., Stenglein M.D., Spencer T.E., Bouma G.J., Anthony R.V., Winger Q.A. (2020). Trophectoderm-Specific Knockdown of LIN28 Decreases Expression of Genes Necessary for Cell Proliferation and Reduces Elongation of Sheep Conceptus. Int. J. Mol. Sci..

[46] Kingsley P.D., Palis J. (1994). GRP2 Proteins Contain Both CCHC Zinc Fingers and a Cold Shock Domain. Plant Cell.

[47] Mihailovich M., Militti C., Gabaldón T., Gebauer F. (2010). Eukaryotic Cold Shock Domain Proteins: Highly Versatile Regulators of Gene Expression. BioEssays.

[48] Piskounova E., Polytarchou C., Thornton J.E., Lapierre R.J., Pothoulakis C., Hagan J.P., Iliopoulos D., Gregory R.I. (2011). Lin28A and Lin28B Inhibit Let-7 MicroRNA Biogenesis by Distinct Mechanisms. Cell.

[49] Hafner M., Max K.E.A., Bandaru P., Morozov P., Gerstberger S., Brown M., Molina H., Tuschl T. (2013). Identification of MRNAs Bound and Regulated by Human LIN28 Proteins and Molecular Requirements for RNA Recognition. RNA.

[50] Molenaar J.J., Domingo-Fernández R., Ebus M.E., Lindner S., Koster J., Drabek K., Mestdagh P., Van Sluis P., Valentijn L.J., Van Nes J. (2012). LIN28B Induces Neuroblastoma and Enhances MYCN Levels via Let-7 Suppression. Nat. Genet..

[51] Balzer E., Moss E.G. (2007). Localization of the Developmental Timing Regulator Lin28 to MRNP Complexes, P-Bodies and Stress Granules. RNA Biol..

[52] Childs A.J., Kinnell H.L., He J., Anderson R.A. (2012). LIN28 Is Selectively Expressed by Primordial and Pre-Meiotic Germ Cells in the Human Fetal Ovary. Stem. Cells Dev..

[53] Kawahara H., Okada Y., Imai T., Iwanami A., Mischel P.S., Okano H. (2011). Musashi1 Cooperates in Abnormal Cell Lineage Protein 28 (Lin28)-Mediated Let-7 Family MicroRNA Biogenesis in Early Neural Differentiation. J. Biol. Chem..

[54] Lott K., Cingolani G. (2011). The Importin β Binding Domain as a Master Regulator of Nucleocytoplasmic Transport. Biochim. Biophys. Acta—Mol. Cell Res..

[55] Pumroy R.A., Cingolani G. (2015). Diversification of Importin-α Isoforms in Cellular Trafficking And. Biochem. J..

[56] Nathaniel B., Whiley P.A.F., Miyamoto Y., Loveland K.L. (2022). Importins: Diverse Roles in Male Fertility. Semin. Cell Dev. Biol..

[57] Kim S., Lee H., Han K., Kim S.C., Choi Y., Park S., Bak G., Lee Y., Choi J.K., Kim T. (2015). SET7/9 Methylation of the Pluripotency Factor LIN28A Is a Nucleolar Localization Mechanism That Blocks Let-7 Biogenesis in Human ESCs. Cell Stem. Cell.

[58] Cardoso-Moreira M., Halbert J., Valloton D., Velten B., Chen C., Shao Y., Liechti A., Ascenção K., Rummel C., Ovchinnikova S. (2019). Gene Expression across Mammalian Organ Development. Nature.

[59] Home < Expression Atlas < EMBL-EBI. https://www.ebi.ac.uk/gxa/home.

[60] Felici M., Farini D., Dolci S. (2009). In or Out Stemness: Comparing Growth Factor Signalling in Mouse Embryonic Stem Cells and Primordial Germ Cells. Curr. Stem. Cell Res. Ther..

[61] Kispert A., Gossler A. (2004). Introduction to Early Mouse Development.

[62] Guo F., Yan L., Guo H., Li L., Hu B., Zhao Y., Yong J., Hu Y., Wang X., Wei Y. (2015). The Transcriptome and DNA Methylome Landscapes of Human Primordial Germ Cells. Cell.

[63] De Felici M. (2013). Origin, Migration, and Proliferation of Human Primordial Germ Cells. Oogenesis.

[64] Sonne S.B., Almstrup K., Dalgaard M., Juncker A.S., Edsgard D., Ruban L., Harrison N.J., Schwager C., Abdollahi A., Huber P.E. (2009). Analysis of Gene Expression Profiles of Microdissected Cell Populations Indicates That Testicular Carcinoma in Situ Is an Arrested Gonocyte. Cancer Res..

[65] Buljubašić R., Buljubašić M., Bojanac A.K., Ulamec M., Vlahović M., Ježek D., Bulić-Jakuš F., Sinčić N. (2018). Epigenetics and Testicular Germ Cell Tumors. Gene.

[66] Gaskell T.L., Esnal A., Robinson L.L.L., Anderson R.A., Saunders P.T.K. (2004). Immunohistochemical Profiling of Germ Cells Within the Human Fetal Testis: Identification of Three Subpopulations. Biol. Reprod..

[67] Guo J., Sosa E., Chitiashvili T., Nie X., Rojas E.J., Oliver E., Plath K., Hotaling J.M., Stukenborg J.B., Clark A.T. (2021). Single-Cell Analysis of the Developing Human Testis Reveals Somatic Niche Cell Specification and Fetal Germline Stem Cell Establishment. Cell Stem. Cell.

[68] Li L., Dong J., Yan L., Yong J., Liu X., Hu Y., Fan X., Wu X., Guo H., Wang X. (2017). Single-Cell RNA-Seq Analysis Maps Development of Human Germline Cells and Gonadal Niche Interactions. Cell Stem. Cell.

[69] Picut C.A., Ziejewski M.K., Stanislaus D. (2018). Comparative Aspects of Pre- and Postnatal Development of the Male Reproductive System. Birth Defects Res..

[70] Shinoda G., De Soysa T.Y., Seligson M.T., Yabuuchi A., Fujiwara Y., Huang P.Y., Hagan J.P., Gregory R.I., Moss E.G., Daley G.Q. (2013). Lin28a Regulates Germ Cell Pool Size and Fertility. Stem Cells.

[71] Hammoud S.S., Low D.H.P., Yi C., Lee C.L., Oatley J.M., Payne C.J., Carrell D.T., Guccione E., Cairns B.R. (2015). Transcription and Imprinting Dynamics in Developing Postnatal Male Germline Stem Cells. Genes Dev..

[72] Gaytan F., Sangiao-Alvarellos S., Manfredi-Lozano M., García-Galiano D., Ruiz-Pino F., Romero-Ruiz A., León S., Morales C., Cordido F., Pinilla L. (2013). Distinct Expression Patterns Predict Differential Roles of the Mirna-Binding Proteins, Lin28 and Lin28b, in the Mouse Testis: Studies during Postnatal Development and in a Model of Hypogonadotropic Hypogonadism. Endocrinology.

[73] Sohni A., Tan K., Song H.W., Burow D., de Rooij D.G., Laurent L., Hsieh T.C., Rabah R., Hammoud S.S., Vicini E. (2019). The Neonatal and Adult Human Testis Defined at the Single-Cell Level. Cell Rep..

[74] Yan W., Ma L., Burns K.H., Matzuk M.M. (2003). HILS1 Is a Spermatid-Specific Linker Histone H1-like Protein Implicated in Chromatin Remodeling during Mammalian Spermiogenesis. Proc. Natl. Acad. Sci. USA.

[75] Tanaka H., Baba T. (2005). Gene Expression in Spermiogenesis. Cell. Mol. Life Sci..

[76] Jun-Hao E.T., Gupta R.R., Shyh-Chang N. (2016). Lin28 and Let-7 in the Metabolic Physiology of Aging. Trends Endocrinol. Metab..

[77] Corre C., Shinoda G., Zhu H., Cousminer D.L., Crossman C., Bellissimo C., Goldenberg A., Daley G.Q., Palmert M.R. (2016). Sex-Specific Regulation of Weight and Puberty by the Lin28/Let-7 Axis. J. Endocrinol..

[78] Sreejith P., Jang W., To V., Jo Y.H., Biteau B., Kim C. (2019). Lin28 Is a Critical Factor in the Function and Aging of Drosophila Testis Stem Cell Niche. Aging.

[79] Wang D., Hou L., Nakamura S., Su M., Li F., Chen W., Yan Y., Green C.D., Chen D., Zhang H. (2017). LIN-28 Balances Longevity and Germline Stem Cell Number in Caenorhabditis Elegans through Let-7/AKT/DAF-16 Axis. Aging Cell.

[80] Leitch H.G., Smith A. (2013). The Mammalian Germline as a Pluripotency Cycle. Development.

[81] Heo I., Joo C., Kim Y.K., Ha M., Yoon M.J., Cho J., Yeom K.H., Han J., Kim V.N. (2009). TUT4 in Concert with Lin28 Suppresses MicroRNA Biogenesis through Pre-MicroRNA Uridylation. Cell.

[82] Jin J., Jing W., Lei X.X., Feng C., Peng S., Boris-Lawrie K., Huang Y. (2011). Evidence That Lin28 Stimulates Translation by Recruiting RNA Helicase A to Polysomes. Nucleic Acids Res..

[83] Wilbert M.L., Huelga S.C., Kapeli K., Stark T.J., Liang T.Y., Chen S.X., Yan B.Y., Nathanson J.L., Hutt K.R., Lovci M.T. (2012). LIN28 Binds Messenger RNAs at GGAGA Motifs and Regulates Splicing Factor Abundance. Mol. Cell.

[84] Polesskaya A., Cuvellier S., Naguibneva I., Duquet A., Moss E.G., Harel-Bellan A. (2007). Lin-28 Binds IGF-2 MRNA and Participates in Skeletal Myogenesis by Increasing Translation Efficiency. Genes Dev..

[85] Bhuiyan M.I.H., Lee J.H., Kim S.Y., Cho K.O. (2013). Expression of Exogenous LIN28 Contributes to Proliferation and Survival of Mouse Primary Cortical Neurons in Vitro. Neuroscience.

[86] Bateman J.M., McNeill H. (2006). Insulin/IGF Signalling in Neurogenesis. Cell. Mol. Life Sci..

[87] Zha J., Lackner M.R. (2010). Targeting the Insulin-like Growth Factor Receptor-1R Pathway for Cancer Therapy. Clin. Cancer Res..

[88] Balzer E., Heine C., Jiang Q., Lee V.M., Moss E.G. (2010). LIN28 Alters Cell Fate Succession and Acts Independently of the Let-7 MicroRNA during Neurogliogenesis In Vitro. Development.

[89] Zhu H., Ng S.C., Segr A.V., Shinoda G., Shah S.P., Einhorn W.S., Takeuchi A., Engreitz J.M., Hagan J.P., Kharas M.G. (2011). The Lin28/Let-7 Axis Regulates Glucose Metabolism. Cell.

[90] Zhang Y., Williams P.R., Jacobi A., Wang C., Goel A., Hirano A.A., Brecha N.C., Kerschensteiner D., He Z. (2019). Elevating Growth Factor Responsiveness and Axon Regeneration by Modulating Presynaptic Inputs. Neuron.

[91] Bingsen X.U., Zhang K., Huang Y. (2009). Lin28 Modulates Cell Growth and Associates with a Subset of Cell Cycle Regulator MRNAs in Mouse Embryonic Stem Cells. RNA.

[92] Xu B., Huang Y. (2009). Histone H2a MRNA Interacts with Lin28 and Contains a Lin28-Dependent Posttranscriptional Regulatory Element. Nucleic Acids Res..

[93] Qiu C., Ma Y., Wang J., Peng S., Huang Y. (2009). Lin28-Mediated Post-Transcriptional Regulation of Oct4 Expression in Human Embryonic Stem Cells. Nucleic Acids Res..

[94] Cho J., Chang H., Kwon S.C., Kim B., Kim Y., Choe J., Ha M., Kim Y.K., Kim V.N. (2012). LIN28A Is a Suppressor of ER-Associated Translation in Embryonic Stem Cells. Cell.

[95] Peng S., Chen L.L., Lei X.X., Yang L., Lin H., Carmichael G.G., Huang Y. (2011). Genome-Wide Studies Reveal That Lin28 Enhances the Translation of Genes Important for Growth and Survival of Human Embryonic Stem Cells. Stem. Cells.

[96] Wang M., Yu L., Wang S., Yang F., Wang M., Li L., Wu X. (2020). LIN28A Binds to Meiotic Gene Transcripts and Modulates Their Translation in Male Germ Cells. J. Cell Sci..

[97] Graf R., Munschauer M., Mastrobuoni G., Mayr F., Heinemann U., Kempa S., Rajewsky N., Landthaler M. (2013). Identification of LIN28B-Bound MRNAs Reveals Features of Target Recognition and Regulation. RNA Biol..

[98] Shinoda G., Shyh-Chang N., Yvanka De Soysa T., Zhu H., Seligson M.T., Shah S.P., Abo-Sido N., Yabuuchi A., Hagan J.P., Gregory R.I. (2013). Fetal Deficiency of Lin28 Programs Life-Long Aberrations in Growth and Glucose Metabolism. Stem. Cells.

[99] Mayr F., Schütz A., Döge N., Heinemann U. (2012). The Lin28 Cold-Shock Domain Remodels Pre-Let-7 MicroRNA. Nucleic Acids Res..

[100] Wang Y., Hu X., Greshock J., Shen L., Yang X. (2012). Genomic DNA Copy-Number Alterations of the Let-7 Family in Human Cancers. PLoS ONE.

[101] Johnson S.M., Grosshans H., Shingara J., Byrom M., Jarvis R., Cheng A., Labourier E., Reinert K.L., Brown D., Slack F.J. (2005). RAS Is Regulated by the Let-7 MicroRNA Family. Cell.

[102] Lee Y.S., Dutta A. (2007). The Tumor Suppressor MicroRNA Let-7 Represses the HMGA2 Oncogene. Genes Dev..

[103] Worringer K.A., Rand T.A., Hayashi Y., Sami S., Takahashi K., Tanabe K., Narita M., Srivastava D., Yamanaka S. (2014). The Let-7/LIN-41 Pathway Regulates Reprogramming to Human Induced Pluripotent Stem Cells by Controlling Expression of Prodifferentiation Genes. Cell Stem. Cell.

[104] Newman M.A., Thomson J.M., Hammond S.M. (2008). Lin-28 Interaction with the Let-7 Precursor Loop Mediates Regulated MicroRNA Processing. RNA.

[105] Martinez N.J., Gregory R.I. (2010). MicroRNA Gene Regulatory Pathways in the Establishment and Maintenance of ESC Identity. Cell Stem. Cell.

[106] Frost R.J.A., Olson E.N. (2011). Control of Glucose Homeostasis and Insulin Sensitivity by the Let-7 Family of MicroRNAs. Proc. Natl. Acad. Sci. USA.

[107] King C.E., Wang L., Winograd R., Madison B.B., Mongroo P.S., Johnstone C.N., Rustgi A.K. (2011). LIN28B Fosters Colon Cancer Migration, Invasion and Transformation through Let-7-Dependent and-Independent Mechanisms. Oncogene.

[108] Lu L., Katsaros D., Shaverdashvili K., Qian B., Wu Y., de la Longrais I.A.R., Preti M., Menato G., Yu H. (2009). Pluripotent Factor Lin-28 and Its Homologue Lin-28b in Epithelial Ovarian Cancer and Their Associations with Disease Outcomes and Expression of Let-7a and IGF-II. Eur. J. Cancer.

[109] Sakurai M., Miki Y., Masuda M., Hata S., Shibahara Y., Hirakawa H., Suzuki T., Sasano H. (2012). LIN28: A Regulator of Tumor-Suppressing Activity of Let-7 MicroRNA in Human Breast Cancer. J. Steroid Biochem. Mol. Biol..

[110] Cao D., Allan R.W., Cheng L., Peng Y., Guo C.C., Dahiya N., Akhi S., Li J. (2011). RNA-Binding Protein LIN28 Is a Marker for Testicular Germ Cell Tumors. Hum. Pathol..

[111] West J.A., Viswanathan S.R., Yabuuchi A., Cunniff K., Takeuchi A., Park I.H., Sero J.E., Zhu H., Perez-Atayde A., Frazier A.L. (2009). A Role for Lin28 in Primordial Germ-Cell Development and Germ-Cell Malignancy. Nature.

[112] Hamano R., Miyata H., Yamasaki M., Sugimura K., Tanaka K., Kurokawa Y., Nakajima K., Takiguchi S., Fujiwara Y., Mori M. (2012). High Expression of Lin28 Is Associated with Tumour Aggressiveness and Poor Prognosis of Patients in Oesophagus Cancer. Br. J. Cancer.

[113] Hovestadt V., Jones D.T.W., Picelli S., Wang W., Kool M., Northcott P.A., Sultan M., Stachurski K., Ryzhova M., Warnatz H.J. (2014). Decoding the Regulatory Landscape of Medulloblastoma Using DNA Methylation Sequencing. Nature.

[114] Manier S., Powers J.T., Sacco A., Glavey S.V., Huynh D., Reagan M.R., Salem K.Z., Moschetta M., Shi J., Mishima Y. (2017). The LIN28B/Let-7 Axis Is a Novel Therapeutic Pathway in Multiple Myeloma.

[115] Zhou J., Chan Z.L., Bi C., Lu X., Chong P.S.Y., Chooi J.Y., Cheong L.L., Liu S.C., Ching Y.Q., Zhou Y. (2017). LIN28B Activation by PRL-3 Promotes Leukemogenesis and a Stem Cell-like Transcriptional Program in AML. Mol. Cancer Res..

[116] Ma X., Li C., Sun L., Huang D., Li T., He X., Wu G., Yang Z., Zhong X., Song L. (2014). Lin28/Let-7 Axis Regulates Aerobic Glycolysis and Cancer Progression via PDK1. Nat. Commun..

[117] Yuko A.E., Carvalho Rigaud V.O., Kurian J., Lee J.H., Kasatkin N., Behanan M., Wang T., Luchesse A.M., Mohsin S., Koch W.J. (2021). LIN28a Induced Metabolic and Redox Regulation Promotes Cardiac Cell Survival in the Heart after Ischemic Injury. Redox Biol..

[118] Mayr F., Heinemann U. (2013). Mechanisms of Lin28-Mediated MiRNA and MRNA Regulation-a Structural and Functional Perspective. Int. J. Mol. Sci..

[119] Loughlin F.E., Gebert L.F.R., Towbin H., Brunschweiger A., Hall J., Allain F.H.T. (2012). Structural Basis of Pre-Let-7 MiRNA Recognition by the Zinc Knuckles of Pluripotency Factor Lin28. Nat. Struct. Mol. Biol..

[120] Piskounova E., Viswanathan S.R., Janas M., LaPierre R.J., Daley G.Q., Sliz P., Gregory R.I. (2008). Determinants of MicroRNA Processing Inhibition by the Developmentally Regulated RNA-Binding Protein Lin28. J. Biol. Chem..

[121] Hagan J.P., Piskounova E., Gregory R.I. (2009). Lin28 Recruits the TUTase Zcchc11 to Inhibit Let-7 Maturation in Mouse Embryonic Stem Cells. Nat. Struct. Mol. Biol..

[122] Heo I., Joo C., Cho J., Ha M., Han J., Kim V.N. (2008). Lin28 Mediates the Terminal Uridylation of Let-7 Precursor MicroRNA. Mol. Cell.

[123] Thornton J.E., Chang H.M., Piskounova E., Gregory R.I. (2012). Lin28-Mediated Control of Let-7 MicroRNA Expression by Alternative TUTases Zcchc11 (TUT4) and Zcchc6 (TUT7). RNA.

[124] Wang L., Nam Y., Lee A.K., Yu C., Roth K., Chen C., Ransey E.M., Sliz P. (2017). LIN28 Zinc Knuckle Domain Is Required and Sufficient to Induce Let-7 Oligouridylation. Cell Rep..

[125] Kolenda T., Przybyla W., Teresiak A., Mackiewicz A., Lamperska K.M. (2014). The Mystery of Let-7d—A Small RNA with Great Power. Wspolczesna Onkol..

[126] Ustianenko D., Chiu H.S., Treiber T., Weyn-Vanhentenryck S.M., Treiber N., Meister G., Sumazin P., Zhang C. (2018). LIN28 Selectively Modulates a Subclass of Let-7 MicroRNAs. Mol. Cell.

[127] Xu M., Bian S., Li J., He J., Chen H., Ge L., Jiao Z., Zhang Y., Peng W., Du F. (2016). MeCP2 Suppresses LIN28A Expression via Binding to Its Methylated-CpG Islands in Pancreatic Cancer Cells. Oncotarget.

[128] Xu J., Zhou Y., Yang J., Gu Y., Zhang E., Yuan W., Wang C., Jin G., Ma H., Hu Z. (2022). Hypomethylation-Activated Cancer-Testis Gene LIN28B Promotes Cell Proliferation and Metastasis in Gastric Cancer. Gene.

[129] Wang L.X., Wang J., Qu T.T., Zhang Y., Shen Y.F. (2014). Reversible Acetylation of Lin28 Mediated by PCAF and SIRT1. Biochim. Biophys. Acta—Mol. Cell Res..

[130] Tsanov K.M., Pearson D.S., Wu Z., Han A., Triboulet R., Seligson M.T., Powers J.T., Osborne J.K., Kane S., Gygi S.P. (2017). LIN28 Phosphorylation by MAPK/ERK Couples Signalling to the Post-Transcriptional Control of Pluripotency. Nat. Cell Biol..

[131] Haq S., Das S., Kim D.H., Chandrasekaran A.P., Hong S.H., Kim K.S., Ramakrishna S. (2019). The Stability and Oncogenic Function of LIN28A Are Regulated by USP28. Biochim. Biophys. Acta—Mol. Basis Dis..

[132] Kim C.W., Vo M.T., Kim H.K., Lee H.H., Yoon N.A., Lee B.J., Min Y.J., Joo W.D., Cha H.J., Park J.W. (2012). Ectopic Over-Expression of Tristetraprolin in Human Cancer Cells Promotes Biogenesis of Let-7 by down-Regulation of Lin28. Nucleic Acids Res..

[133] Lee J.Y., Kim H.J., Yoon N.A., Lee W.H., Min Y.J., Ko B.K., Lee B.J., Lee A., Cha H.J., Cho W.J. (2013). Tumor Suppressor P53 Plays a Key Role in Induction of Both Tristetraprolin and Let-7 in Human Cancer Cells. Nucleic Acids Res..

[134] Chen T., Chen C., Wu H., Chen X., Xie R., Wang F., Sun H., Chen L. (2021). Overexpression of P53 Accelerates Puberty in High-Fat Diet–Fed Mice through Lin28/Let-7 System. Exp. Biol. Med..

[135] Tocchini C., Ciosk R. (2015). TRIM-NHL Proteins in Development and Disease. Semin. Cell Dev. Biol..

[136] Lipkowitz S., Weissman A.M. (2011). RINGs of Good and Evil: RING Finger Ubiquitin Ligases at the Crossroads of Tumour Suppression and Oncogenesis. Nat. Rev. Cancer.

[137] Lee S.H., Cho S., Sun Kim M., Choi K., Cho J.Y., Gwak H.S., Kim Y.J., Yoo H., Lee S.H., Park J.B. (2014). The Ubiquitin Ligase Human TRIM71 Regulates Let-7 MicroRNA Biogenesis via Modulation of Lin28B Protein. Biochim. Biophys. Acta—Gene Regul. Mech..

[138] Yin J., Kim T.-H., Park N., Shin D., Choi H.I., Cho S., Park J.B., Kim J.H., Bae J. (2016). TRIM71 Suppresses Tumorigenesis via Modulation of Lin28B-Let-7-HMGA2 Signaling. Oncotarget.

[139] Singh R., Junghare V., Hazra S., Singh U., Sengar G.S., Raja T.V., Kumar S., Tyagi S., Das A.K., Kumar A. (2019). Database on Spermatozoa Transcriptogram of Catagorised Frieswal Crossbred (Holstein Friesian X Sahiwal) Bulls. Theriogenology.

[140] Zhong X., Li N., Liang S., Huang Q., Coukos G., Zhang L. (2010). Identification of MicroRNAs Regulating Reprogramming Factor LIN28 in Embryonic Stem Cells and Cancer Cells. J. Biol. Chem..

[141] Wu L., Belasco J.G. (2005). Micro-RNA Regulation of the Mammalian. Society.

[142] Li X., Zhang J., Gao L., McClellan S., Finan M.A., Butler T.W., Owen L.B., Piazza G.A., Xi Y. (2012). MiR-181 Mediates Cell Differentiation by Interrupting the Lin28 and Let-7 Feedback Circuit. Cell Death Differ..

[143] Murray M.J., Saini H.K., Siegler C.A., Hanning J.E., Barker E.M., Van Dongen S., Ward D.M., Raby K.L., Groves I.J., Scarpini C.G. (2013). LIN28 Expression in Malignant Germ Cell Tumors Downregulates Let-7 and Increases Oncogene Levels. Cancer Res..

[144] Balzeau J., Menezes M.R., Cao S., Hagan J.P. (2017). The LIN28/Let-7 Pathway in Cancer. Front. Genet..

[145] Murray M.J., Nicholson J.C., Coleman N. (2015). Biology of Childhood Germ Cell Tumours, Focussing on the Significance of MicroRNAs. Andrology.

[146] Chakraborty P., Buaas F.W., Sharma M., Snyder E., Rooij D.G., Braun R.E., William Buaas F., De Rooij D.G. (2014). LIN28A Marks the Spermatogonial Progenitor Population and Regulates Its Cyclic Expansion. Stem. Cells.

[147] Werler S., Demond H., Damm O.S., Ehmcke J., Middendorff R., Gromoll J., Wistuba J. (2014). Germ Cell Loss Is Associated with Fading Lin28a Expression in a Mouse Model for Klinefelter’s Syndrome. Reproduction.

[148] Zheng K., Wu X., Kaestner K.H., Wang P.J. (2009). The Pluripotency Factor LIN28 Marks Undifferentiated Spermatogonia in Mouse. BMC Dev. Biol..

[149] Sèdes L., Desdoits-Lethimonier C., Rouaisnel B., Holota H., Thirouard L., Lesne L., Damon-Soubeyrand C., Martinot E., Saru J.P., Mazaud-Guittot S. (2018). Crosstalk between BPA and FXRα Signaling Pathways Lead to Alterations of Undifferentiated Germ Cell Homeostasis and Male Fertility Disorders. Stem. Cell Rep..

[150] Brieño-Enríquez M.A., García-López J., Cárdenas D.B., Guibert S., Cleroux E., Děd L., Hourcade J.D.D., Pěknicová J., Weber M., Del Mazo J. (2015). Exposure to Endocrine Disruptor Induces Transgenerational Epigenetic Deregulation of MicroRNAs in Primordial Germ Cells. PLoS ONE.

[151] Rochester J.R. (2013). Bisphenol A and Human Health: A Review of the Literature. Reprod. Toxicol..

[152] Anway M.D., Cupp A.S., Uzumcu N., Skinner M.K. (2005). Toxicology: Epigenetic Transgenerational Actions of Endocrine Disruptors and Male Fertility. Science.

[153] Matsuura I., Saitoh T., Ashina M., Wako Y., Iwata H., Toyota N., Ishizuka Y., Namiki M., Hoshino N., Tsuchitani M. (2005). Evaluation of a Two-Generation Reproduction Toxicity Study Adding Endopoints to Detect Endocrine Disrupting Activity Using Vinclozolin. J. Toxicol. Sci..

[154] Spiller C.M., Bowles J. (2017). Germ Cell Neoplasia in Situ: The Precursor Cell for Invasive Germ Cell Tumors of the Testis. Int. J. Biochem. Cell Biol..

[155] Lv K., Liu L., Wang L., Yu J., Liu X., Cheng Y., Dong M., Teng R., Wu L., Fu P. (2012). Lin28 Mediates Paclitaxel Resistance by Modulating P21, Rb and Let-7a MiRNA in Breast Cancer Cells. PLoS ONE.

[156] Wang L., Yuan C., Lv K., Xie S., Fu P., Liu X., Chen Y., Qin C., Deng W., Hu W. (2013). Lin28 Mediates Radiation Resistance of Breast Cancer Cells via Regulation of Caspase, H2AX and Let-7 Signaling. PLoS ONE.

[157] Jee-Sun Oh M.S., Jae-Jin Kim B.S., Ju-Yeon Byun M.S., In-Ah Kim M.D. (2010). Lin28-let7 modulates radiosensitivity of human cancer cells with activation of k-ras. Int. J. Radiat. Oncol. * Biol. * Phys..

[158] Qin R., Zhou J., Chen C., Xu T., Yan Y., Ma Y., Zheng Z., Shen Y., Lu Y., Fu D. (2014). LIN28 Is Involved in Glioma Carcinogenesis and Predicts Outcomes of Glioblastoma Multiforme Patients. PLoS ONE.

[159] Kong D., Banerjee S., Ahmad A., Li Y., Wang Z., Sethi S., Sarkar F.H. (2010). Epithelial to Mesenchymal Transition Is Mechanistically Linked with Stem Cell Signatures in Prostate Cancer Cells. PLoS ONE.

[160] Zhang H., Zong Y., Qiu G., Jia R., Xu X., Wang F., Wu D. (2018). Silencing Lin28 Promotes Apoptosis in Colorectal Cancer Cells by Upregulating Let-7c Targeting of Antiapoptotic BCL2L1. Mol. Med. Rep..

[161] Chen Y., Xie C., Zheng X., Nie X., Wang Z., Liu H., Zhao Y. (2019). LIN28/Let-7/PD-L1 Pathway as a Target for Cancer Immunotherapy. Cancer Immunol. Res..

[162] Sikora K., Evan G., Stewart J., Watson J.V. (1985). Detection of the C-Myc Oncogene Product in Testicular Cancer. Br. J. Cancer.

[163] Roos M., Pradère U., Ngondo R.P., Behera A., Allegrini S., Civenni G., Zagalak J.A., Marchand J.R., Menzi M., Towbin H. (2016). A Small-Molecule Inhibitor of Lin28. ACS Chem. Biol..

[164] Wang L., Rowe R.G., Jaimes A., Yu C., Nam Y., Pearson D.S., Zhang J., Xie X., Marion W., Heffron G.J. (2018). Small-Molecule Inhibitors Disrupt Let-7 Oligouridylation and Release the Selective Blockade of Let-7 Processing by LIN28. Cell Rep..

[165] Chen H., Sa G., Li L., He S., Wu T. (2021). In Vitro and in Vivo Synergistic Anti-Tumor Effect of LIN28 Inhibitor and Metformin in Oral Squamous Cell Carcinoma. Eur. J. Pharmacol..

[166] Rhee Y.H., Kim T.H., Jo A.Y., Chang M.Y., Park C.H., Kim S.M., Song J.J., Oh S.M., Yi S.H., Kim H.H. (2016). LIN28A Enhances the Therapeutic Potential of Cultured Neural Stem Cells in a Parkinson’s Disease Model. Brain.

[167] Nathan F.M., Ohtake Y., Wang S., Jiang X., Sami A., Guo H., Zhou F.Q., Li S. (2020). Upregulating Lin28a Promotes Axon Regeneration in Adult Mice with Optic Nerve and Spinal Cord Injury. Mol. Ther..

